# Adult-onset deficiency of β-klotho in the liver reveals a novel role in lipid homeostasis

**DOI:** 10.1016/j.molmet.2026.102443

**Published:** 2026-09-22

**Authors:** Alexandra S. Aaldijk, Cristy R.C. Verzijl, Milaine V. Hovingh, Nicolette C.A. Huijkman, Marieke Smit, Niels J. Kloosterhuis, Niels L. Mulder, Rick Havinga, Albert Gerding, Trijnie Bos, Mirjam Koster, Mariska de Graaf, Justina C. Wolters, Bart van de Sluis, Dicky Struik, Johan W. Jonker

**Affiliations:** 1 Department of Pediatrics, University of Groningen, University Medical Center Groningen, Hanzeplein 1, 9700 RB Groningen, the Netherlands; 2 Interfaculty Mass Spectrometry Center, Antonius Deusinglaan 1, 9713 AV Groningen, the Netherlands; 3 Transgenic Mouse Clinic for Aging Research, Antonius Deusinglaan 1, 9713 AV Groningen, the Netherlands

**Keywords:** MASLD, FGF19, FGF21, Liver metabolism, Bile acids, Lipoprotein, VLDL, Somatic

## Abstract

**Background:**

β-klotho (KLB), the obligate co-receptor for fibroblast growth factor (FGF) 19 and 21 signalling, has emerged as a therapeutic target for metabolic disease. Current understanding of KLB function is based largely on germline knockout mouse models, where developmental abnormalities obscure its function in adult physiology. Here, we used somatic hepatocyte-specific gene editing in adult mice to define hepatic KLB function.

**Methods:**

Hepatocyte-specific KLB deficiency was induced by retro-orbital delivery of adeno-associated virus particles containing guide RNAs targeting *Klb* in adult male mice expressing Cas9 in the liver. Following transduction, mice were maintained on a 4-week chow diet or a 12-week high-fat diet, prior to comprehensive metabolic and biochemical phenotyping.

**Results:**

Adult-onset hepatic KLB deficiency did not alter body weight or composition, indicating that reduced bodyweight in germline models reflects developmental effects. In line with previous studies, KLB deficiency resulted in derepressed bile acid synthesis, elevated faecal bile acid excretion, and a shift towards a more hydrophobic bile acid pool. Importantly, we also uncovered a role for hepatic KLB in regulating lipid homeostasis. Chow-fed KLB-deficient mice exhibited reduced circulating very-low-density lipoprotein levels (VLDL) with marked hepatic triglyceride accumulation, suggesting altered VLDL metabolism as a potential mechanism driving steatosis.

**Conclusions:**

Using an adult-onset liver-specific knockout model, we disentangled KLB function from developmental confounders and established KLB as a critical regulator of adult lipid and lipoprotein homeostasis. Our study redefines the physiological role of KLB in hepatic metabolism and has important implications for the interpretation of FGF19/KLB-directed therapies for metabolic diseases.

## Highlights

• Current understanding of KLB mainly relies on germline knockout mouse models.
• Somatic liver-specific KLB knockout eliminates confounding effects of development.
• Somatic hepatic KLB loss identifies novel role for KLB in lipid metabolism.
• Hepatic KLB deficiency affects lipoprotein metabolism by lowering plasma VLDL.
• This research refines understanding of FGF19/KLB-targeted therapy in liver disease.

## Graphical abstract

**Figure:**
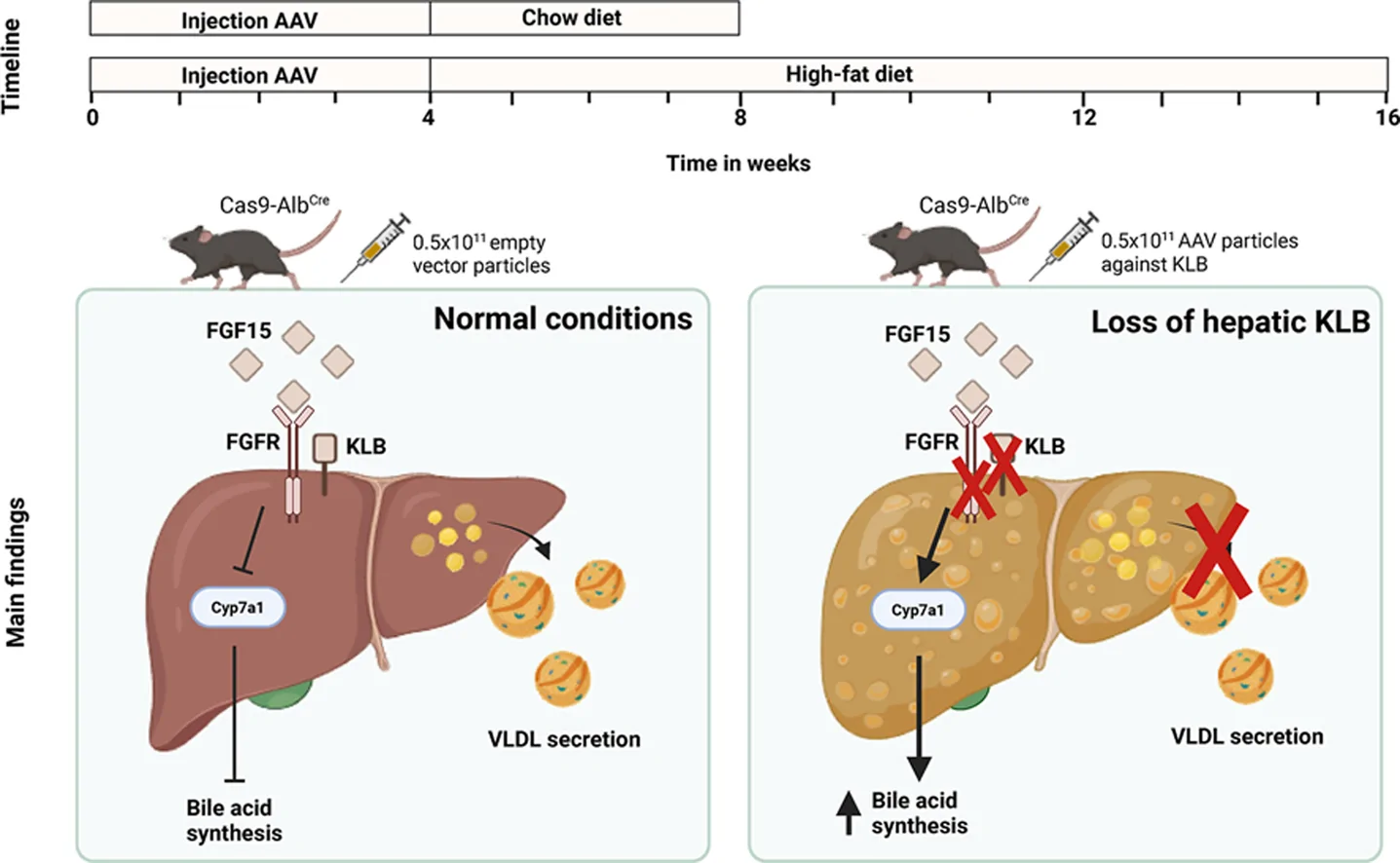

## 1 Introduction

Fibroblast growth factors (FGF)19 and FGF21 have emerged as crucial players in glucose and lipid metabolism. FGF19 is predominantly secreted by ileal enterocytes in response to bile acids and acts to suppress hepatic bile acid synthesis and to promote glycogen storage, thereby regulating metabolic adaptation after a meal [1,2]. FGF21 is secreted by the liver and acts as an endocrine regulator of lipid oxidation and energy expenditure, particularly during fasting or ketogenesis [3]. Given their critical roles in metabolism, FGF19/21-based drugs have therapeutic potential in various liver diseases, including metabolic dysfunction-associated steatotic liver disease (MASLD) and cholestatic liver diseases [4]. A recent meta-analysis revealed that FGF21-based interventions outperformed resmetirom and GLP-1 agonists in fibrosis improvement, while simultaneously exhibiting fewer adverse effects [5]. Both FGF19 and FGF21 require the co-receptor beta-klotho (KLB) to facilitate the binding between FGF19/21 and their cognate FGF receptors (FGFR1-4), which enables downstream signalling that regulates key aspects of metabolic homeostasis [6,7]. As KLB also has a more restricted tissue distribution compared to the ubiquitously expressed FGFRs, KLB potentially has a superior therapeutic profile. Hence, a better understanding of KLB function in the liver is of significant interest to improve therapeutic strategies.

Previous studies have mainly used germline gene editing to generate whole-body or tissue-specific KLB knockout mouse models and investigate the physiological functions of KLB. Mice with a germline deletion of KLB display growth retardation (reduced body and femur length), liver inflammation and fibrogenesis, and metabolic dysregulation, as demonstrated by an increased bile acid synthesis rate and shift in bile acid composition as well as faster glucose turnover [[8], [9], [10]]. Moreover, KLB-derived changes in bile acid composition can in turn influence gut microbiota and thermogenic activity [11]. Germline loss of KLB was also linked to insulin resistance, dyslipidaemia, and an increased susceptibility to hepatic steatosis, possibly due to disrupted FGF-mediated regulation of lipid metabolism, thereby mimicking features of MASLD [12,13]. Thus, these findings highlight the key role of KLB in metabolic health and suggest that its dysfunction may contribute to MASLD or cholestatic liver disease.

While these developmental models have provided valuable insights into the function of KLB, the use of germline gene editing can lead to severe developmental defects. The International Mouse Phenotyping Consortium (IMPC) reports that whole-body knockout of KLB in mice leads to abnormal placenta size and morphology, and reduced liver weight and body weight [14,15]. These defects not only hamper the understanding of the physiological role of KLB in metabolism but also the pharmacodynamic response to FGF19- and FGF21-based therapies [16]. As the function of KLB in adult physiology is insufficiently characterized, this poses a gap in our understanding of its role in metabolic regulation. To overcome these limitations and dissect the function of hepatic KLB in adult metabolic regulation independently of developmental perturbations, we employed a liver-specific Cas9-mediated somatic gene editing strategy in adult mice. As the liver is a major site of KLB expression [17], we delivered KLB-targeting sgRNAs to liver-specific Cas9-expressing adult male mice using adeno-associated virus particles. This strategy enables investigation of the role of hepatic KLB in metabolic homeostasis during adulthood, without the confounding influence on development.

## 2 Results

### 2.1 Generation of hepatocyte-specific KLB-deficient mice

To obtain insight into the physiological role of KLB in hepatic metabolism, male liver-specific *Klb* knockout mice (*Klb*^HepKO^) were generated by targeting exon 1 of the mouse *Klb* gene using CRISPR/Cas9 somatic gene editing (Figure 1A). Transgenic Cas9 mice receiving an empty AAV vector were used as controls (EV^Hep^). Effective Cas9-mediated gene editing was validated using qPCR primer pairs that anneal within and outside the sgRNA-target sequences (Supplementary table 1), showing an overall gene editing efficiency of 89.7% (Figure 1B) and a 91.5% reduction in hepatic KLB protein levels (Figure 1C, D, Supplementary Figure 1). Hepatocyte-specific deletion of *Klb* did not affect body weight in mice fed a chow or HFD, nor did it result in changes in fat or lean mass (Figure 1E, F) compared to EV^Hep^ mice.

**Figure 1:**
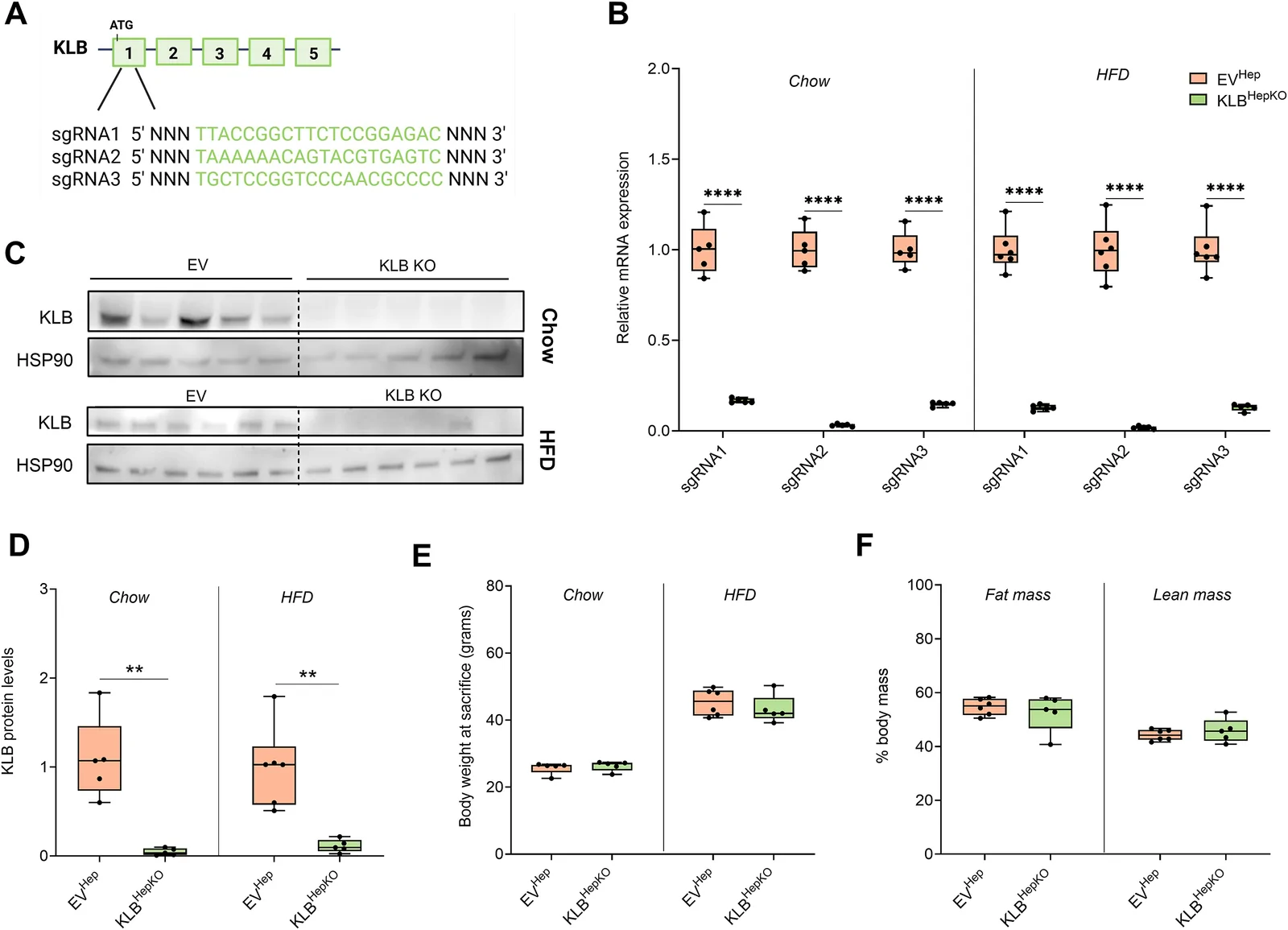
**Liver-specific knockout of KLB does not alter body weight or body composition.** (A) Somatic gene editing strategy to knockout the *Klb* gene using guide RNAs in the livers of Cas9-expressing mice. (B) Gene editing efficacy using qPCR primer pairs for KLB sgRNA 1–3 that anneal within and outside the sgRNA-target sequences (*Chow*; n = 5 for EV^Hep^ and n = 5 for *Klb*^HepKO^, *HFD*; n = 6 for EV^Hep^ and n = 5 for *Klb*^HepKO^), normalized against the U36b4 gene, (C) Hepatic KLB protein levels (*Chow*; n = 5 for EV^Hep^ and n = 5 for *Klb*^HepKO^, *HFD*; n = 6 for EV^Hep^ and n = 5 for *Klb*^HepKO^), (D) Quantified hepatic protein levels of KLB (*Chow*; n = 5 for EV^Hep^ and n = 5 for *Klb*^HepKO^, *HFD*; n = 6 for EV^Hep^ and n = 5 for *Klb*^HepKO^), normalized against HSP90, and (E) Body weight (*Chow*; n = 5 for EV^Hep^ and n = 5 for *Klb*^HepKO^, *HFD*; n = 6 for EV^Hep^ and n = 5 for *Klb*^HepKO^) in EV^Hep^ and *Klb*^HepKO^ mice following 4 weeks of chow diet or 12 weeks of high-fat diet regimen. (F) % fat mass and fat free mass (*Chow*; n = 5 for EV^Hep^ and n = 5 for *Klb*^HepKO^, *HFD*; n = 6 for EV^Hep^ and n = 5 for *Klb*^HepKO^) in EV^Hep^ and *Klb*^HepKO^ mice following 12 weeks of high-fat diet. Data are presented as mean ± 95% CI in Tukey box- and whisker plots with individual datapoints. Statistical significance was determined using nonparametric Mann–Whitney testing for the comparison of two groups and indicated as ∗*P* < 0.05, ∗∗*P* < 0.01, ∗∗∗*P* < 0.001, ∗∗∗∗*P* < 0.0001. EV: empty vector, KO: knockout, KLB: beta-klotho, sgRNA: single guide RNA, HFD: high-fat diet, HSP90: heatshock protein 90.

As CRISPR/Cas9-mediated gene editing can be associated with off-target effects [18], we have generated an independent mouse model to address these concerns. Here, we used an alternative AAV construct containing sgRNAs targeting both exon 1 and 2 of mouse *Klb* rather than our initial AAV construct only targeting exon 1. The phenotypes of these two independent mouse models were very similar, validating the reproducibility of the observed genetic effect and eliminating the presence of any shared off-target artefacts (Supplementary Figure 2A-F).

### 2.2 Loss of hepatic KLB increases bile acid synthesis through elevated *Cyp7a1* levels and induces a more hydrophobic bile acid pool

As KLB is an obligatory co-receptor for FGF15/19 signalling, previous studies using whole-body knockout mouse models described a role for FGF15/FGF19 as well as KLB in the regulation of bile acid metabolism [8,19,20]. First, we confirmed that upon loss of KLB, there is loss of EGR1 protein levels, suggesting indeed the loss of FGF19/21 signalling (Supplementary Figure 3A). In line with these studies, hepatic mRNA expression of *Cyp7a1* and *Cyp8b1*, which encode rate-limiting enzymes in the classical bile acid synthesis pathway, was increased 2.6-fold and 2.2-fold, respectively, in *Klb*^HepKO^ mice compared to EV^Hep^ mice under chow conditions, indicating an increased bile acid synthesis rate (Figure 2A, B). This increase was even more pronounced under HFD conditions, showing a 5.8-fold and 2.4-fold increase, respectively, in *Klb*^HepKO^ mice compared to EV^Hep^ mice (Figure 2A, B). Hepatic expression of *Cyp27a1*, encoding an enzyme involved in the synthesis of bile acids through the alternative pathway, was increased 2.7-fold in chow-fed *Klb*^HepKO^ mice compared to EV^Hep^ mice but not when fed an HFD (Figure 2C). Hepatic *Cyp7b1* mRNA expression, encoding another enzyme in the alternative bile acid synthesis pathway, remained unchanged (Figure 2D). Collectively, these transcriptional changes suggest that hepatic KLB suppresses bile acid synthesis via the classical pathway by reducing the expression of *Cyp7a1* and *Cyp8b1*.

**Figure 2:**
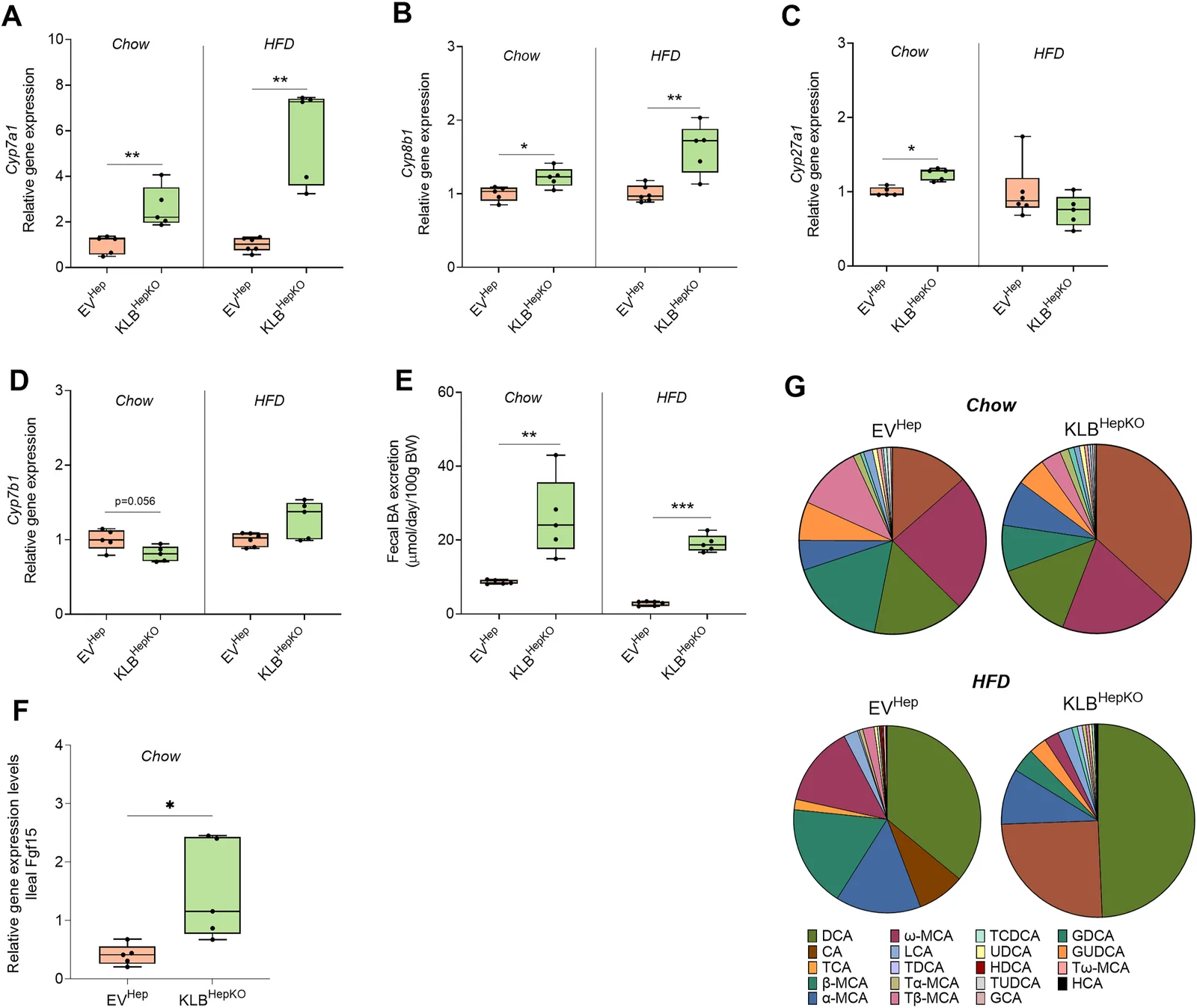
**Hepatic knockout of *Klb* increases bile acid synthesis and faecal bile acid excretion, as well as the faecal bile acid composition.** (A) *Cyp7a1* mRNA expression levels (*Chow*; n = 5 for EV^Hep^ and n = 5 for *Klb*^HepKO^, *HFD*; n = 6 for EV^Hep^ and n = 5 for *Klb*^HepKO^), (B) *Cyp8b1* mRNA expression levels (*Chow*; n = 5 for EV^Hep^ and n = 5 for *Klb*^HepKO^, *HFD*; n = 6 for EV^Hep^ and n = 5 for *Klb*^HepKO^), (C) *Cyp27a1* mRNA expression levels (*Chow*; n = 5 for EV^Hep^ and n = 5 for *Klb*^HepKO^, *HFD*; n = 6 for EV^Hep^ and n = 5 for *Klb*^HepKO^), (D) *Cyp7b1* mRNA expression levels (*Chow*; n = 5 for EV^Hep^ and n = 5 for *Klb*^HepKO^, *HFD*; n = 6 for EV^Hep^ and n = 5 for *Klb*^HepKO^), (E) Faecal bile acid composition (*Chow*; n = 5 for EV^Hep^ and n = 5 for *Klb*^HepKO^, *HFD*; n = 6 for EV^Hep^ and n = 5 for *Klb*^HepKO^), and (F) Faecal bile acid excretion (*Chow*; n = 5 for EV^Hep^ and n = 5 for *Klb*^HepKO^, *HFD*; n = 6 for EV^Hep^ and n = 5 for *Klb*^HepKO^) in *Klb*^HepKO^ mice compared to EV^Hep^ mice following 4 weeks of chow diet or 12 weeks of HFD. (G) Relative ileal *Fgf1*5 mRNA expression levels (*Chow*; n = 5 for EV^Hep^ and n = 5 for *Klb*^HepKO^, *HFD*; n = 6 for EV^Hep^ and n = 5 for *Klb*^HepKO^), and (H) Relative hepatic *Tgr5* mRNA expression levels (*Chow*; n = 5 for EV^Hep^ and n = 5 for *Klb*^HepKO^, *HFD*; n = 6 for EV^Hep^ and n = 5 for *Klb*^HepKO^) in *Klb*^HepKO^ mice compared to EV^Hep^ mice following 4 weeks of chow diet. Data are presented as mean ± 95% CI in Tukey box- and whisker plots with individual datapoints. Statistical significance was determined using nonparametric Mann–Whitney testing for the comparison of two groups and indicated as ∗*P* < 0.05, ∗∗*P* < 0.01, ∗∗∗*P* < 0.001, ∗∗∗∗*P* < 0.0001. EV: empty vector, KO: knockout, KLB: beta-klotho, HFD: high-fat diet, Cyp7a1: cholesterol 7α-hydroxylase, Cyp8b1: cytochrome P450 family 8 subfamily B member 1, Cyp27a1: sterol 27-hydroxylase, Cyp7b1: cytochrome P450 Family 7 Subfamily B Member 1, BA: bile acid, Fgf15: fibroblast growth factor 15, Tgr5: Takeda G protein-coupled receptor 5.

Under steady-state conditions, faecal bile acid excretion reflects the rate of bile acid synthesis. Indeed, in line with increased expression of the bile acid synthesis enzymes, faecal bile acid excretion was increased 2.9-fold in chow-fed and 7.9-fold in HFD-fed *Klb*^HepKO^ mice compared to EV^Hep^ mice (Figure 2E). Despite unaltered hepatic and ileal *Fxr* expression levels, the increased synthesis of bile acids induced transcriptional activation of ileal *Fgf15 (*Figure 2F), suggesting increased bile acids in the ileum and the ileal activation of *Fxr*. Hepatic KLB deficiency also altered faecal BA composition, as reflected by a greater proportion of cholic acid (CA, +14.2%) and deoxycholic acid (DCA, +3.6**%**) at the expense of muricholic acids (MCAs, −16.5%) in chow-fed mice (Figure 2G, Supplementary Table 3 and 4). Under HFD conditions, these effects were more pronounced with a greater proportion of CA and DCA (+16,9% and +13.3%, respectively) at the expense of hydrophilic muricholic acids (−32,4%), resulting in a more hydrophobic faecal bile acid composition in *Klb*^HepKO^ mice compared to EV^Hep^ mice. While total plasma bile acid levels were not affected on chow or HFD (Supplementary Figure 3B), plasma bile acid composition did alter due to hepatic KLB deficiency, showing an increased abundance of taurocholic acid (TCA) (+6.0%) and CA (+4.5%), and a decreased abundance of tauro-β-muricholic acid (Tβ-MCA, −18.8%) when fed a chow diet (Supplementary Figure 3C, Supplementary Table 5 and 6). Similarly, when fed an HFD, hepatic deficiency of KLB resulted in a greater increase in abundance of TCA (+14.4%) and DCA (+5.0%), and a greater decrease in abundance of Tβ-MCA (−22.6%) and Tα-MCA (−5.3%) (Supplementary Table 5 and 6).

The formation of bile is influenced by biliary bile acid secretion. While bile flow and biliary bile acid secretion were unaffected by hepatic KLB deficiency under chow conditions (Figure 3A, B), biliary bile acid composition and hydrophobicity did alter significantly. *Klb*^HepKO^ mice showed a greater proportion of TCA (+14%) and Tα-MCA (+2.4%) in bile at the expense of Tβ-MCA (−19.3%) (Figure 3C, Supplementary Table 7 and 8), resulting in a more hydrophobic bile acid pool as calculated according to Heuman [21](Figure 3D). The altered bile acid composition was also characterized by a 1.8-fold increased ratio of 12α-hydroxylated bile acids (Figure 3E). When fed an HFD, hepatic deficiency of KLB also resulted in a 1.5-fold increased bile flow and a 1.8-fold increased biliary bile acid secretion (Figure 3A, B). Additionally, changes in bile acid composition were even more pronounced under HFD conditions in KLB^HepKO^ mice compared to EV^Hep^ mice, as shown by a strong elevation of TCA (+19,7%) but also TDCA (+11,7%), at the expense of Tβ-MCA (−27.2%) (Figure 3C, Supplementary Table 7 and 8), translating into a markedly increased hydrophobicity index (Figure 3D). The ratio of 12α-hydroxylated bile acids was 4.7-fold increased in KLB^HepKO^ mice compared to EV^Hep^ mice (Figure 3E), showing that bile acid composition changes more greatly upon knockout of *Klb* under HFD conditions. Finally, in line with bile acids driving cholesterol and phospholipid secretion into the bile to prevent hepatocellular damage [22,23], *Klb*^HepKO^ mice on a HFD showed a 1.7-fold greater biliary cholesterol secretion and a 2.2-fold greater biliary phospholipid secretion compared to EV^Hep^ mice (Figure 3F, G).

**Figure 3:**
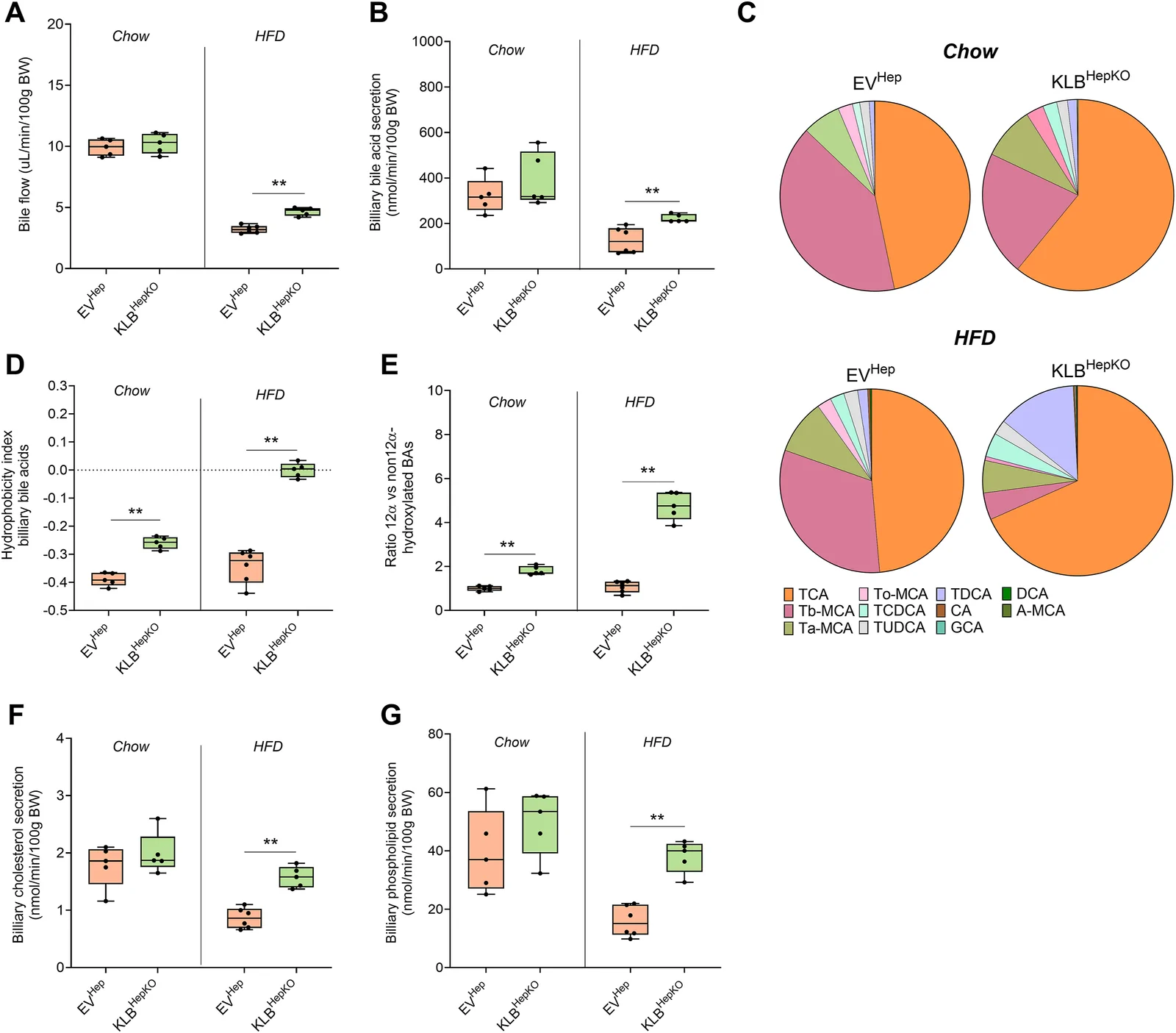
**Loss of hepatic KLB alters biliary bile acid composition, increases the hydrophobicity of the biliary bile acid pool, and enhances biliary cholesterol and phospholipid secretion.** (A) Bile flow (*Chow*; n = 5 for EV^Hep^ and n = 5 for *Klb*^HepKO^, *HFD*; n = 6 for EV^Hep^ and n = 5 for *Klb*^HepKO^), (B) Biliary bile acid secretion (*Chow*; n = 5 for EV^Hep^ and n = 5 for *Klb*^HepKO^, *HFD*; n = 6 for EV^Hep^ and n = 5 for *Klb*^HepKO^), (C) Biliary bile acid composition (*Chow*; n = 5 for EV^Hep^ and n = 5 for *Klb*^HepKO^, *HFD*; n = 6 for EV^Hep^ and n = 5 for *Klb*^HepKO^), (D) Hydrophobicity index (*Chow*; n = 5 for EV^Hep^ and n = 5 for *Klb*^HepKO^, *HFD*; n = 6 for EV^Hep^ and n = 5 for *Klb*^HepKO^), (E) Ratio of 12alpha- and non-12alpha-hydroxylated bile acid (*Chow*; n = 5 for EV^Hep^ and n = 5 for *Klb*^HepKO^, *HFD*; n = 6 for EV^Hep^ and n = 5 for *Klb*^HepKO^), (F) Biliary cholesterol secretion (*Chow*; n = 5 for EV^Hep^ and n = 5 for *Klb*^HepKO^, *HFD*; n = 6 for EV^Hep^ and n = 5 for *Klb*^HepKO^), and (G) Biliary phospholipid secretion (*Chow*; n = 5 for EV^Hep^ and n = 5 for *Klb*^HepKO^, *HFD*; n = 6 for EV^Hep^ and n = 5 for *Klb*^HepKO^) in *Klb*^HepKO^ mice compared to EV^Hep^ mice following 4 weeks of chow diet or 12 weeks of HFD. Data are presented as mean ± 95% CI in Tukey box- and whisker plots with individual datapoints and pie charts. Statistical significance was determined using nonparametric Mann–Whitney testing for the comparison of two groups and indicated as ∗*P* < 0.05, ∗∗*P* < 0.01, ∗∗∗*P* < 0.001, ∗∗∗∗*P* < 0.0001. EV: empty vector, KO: knockout, KLB: beta-klotho, HFD: high-fat diet.

Overall, hepatic KLB deficiency increased bile acid synthesis and excretion and shifted bile acid composition toward a more hydrophobic bile acid pool. Furthermore, the effects of hepatic KLB deficiency on bile acid composition and hydrophobicity were greater under HFD conditions compared to chow conditions.

### 2.3 Loss of hepatic KLB elevates hepatic triglyceride levels while lowering plasma triglyceride levels

As an increased bile acid synthesis affects overall cholesterol turnover, we assessed plasma and hepatic cholesterol levels. However, no changes in plasma or hepatic cholesterol were observed in *Klb*^HepKO^ mice compared to EV^Hep^ mice (Supplementary Figure 3D-E, Figure 4A). Given the prominent role of bile acids in regulating lipid metabolism [24], we further investigated whether plasma triglyceride and non-esterified fatty acid (NEFA) levels were altered upon knockout of *Klb*. While NEFA levels were unaffected under both dietary conditions (Figure 4B), plasma triglyceride levels were decreased by 41.4% in *Klb*^HepKO^ mice compared to EV^Hep^ mice on a chow diet (Figure 4C). Liver H&E staining did not reveal major histological differences between EV^Hep^ and *Klb*^HepKO^ mice (Figure 4D), and there were no differences in liver weight and plasma levels of liver damage markers AST/ALT (Figure 4E, F). Random and fasting blood glucose levels at 8 weeks were also unaltered between HFD-fed EV^Hep^ and *Klb*^HepKO^ mice (Supplementary Figure 3F). Hepatic levels of triglycerides were 37% increased in *Klb*^HepKO^ mice compared to EV^Hep^ mice (Figure 4G), while hepatic phospholipid levels remained unaltered (Figure 4H). In line with the increased hepatic triglyceride levels, Oil-Red-O staining revealed increased fat content in the liver in *Klb*^HepKO^ mice fed a HFD (Figure 4D). At 8 weeks of HFD, plasma triglyceride levels were still decreased by 50% in *Klb*^HepKO^ mice compared to EV^Hep^ mice (Supplementary Figure 3G). However, the effects of hepatic KLB deficiency on plasma and hepatic triglycerides were lost after 12 weeks of HFD (Figure 4C, G). Similar to chow conditions, hepatic cholesterol and phospholipid levels were unaffected in *Klb*^HepKO^ mice.

**Figure 4:**
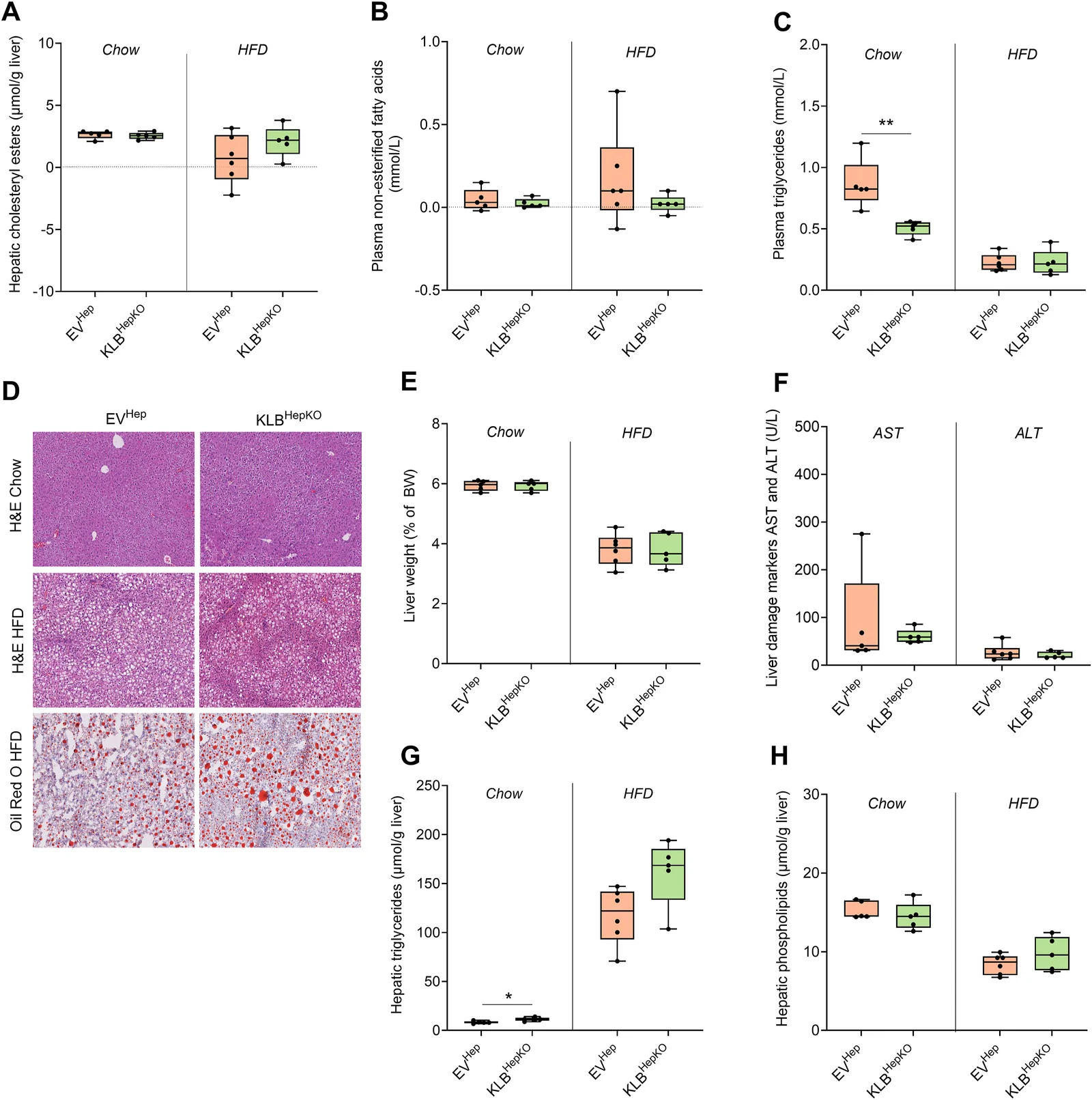
**Loss of hepatic KLB affects hepatic lipid metabolism in chow-fed mice but not HFD-fed mice.** (A) Hepatic cholesteryl ester levels (*Chow*; n = 5 for EV^Hep^ and n = 5 for *Klb*^HepKO^, *HFD*; n = 6 for EV^Hep^ and n = 5 for *Klb*^HepKO^), (B) plasma non-esterified fatty acid levels (*Chow*; n = 5 for EV^Hep^ and n = 5 for *Klb*^HepKO^, *HFD*; n = 6 for EV^Hep^ and n = 5 for *Klb*^HepKO^), (C) plasma triglycerides (*Chow*; n = 5 for EV^Hep^ and n = 5 for *Klb*^HepKO^, *HFD*; n = 6 for EV^Hep^ and n = 5 for *Klb*^HepKO^), (D) H&E and Oil Red O staining, (E) liver weight as a percentage of body weight (*Chow*; n = 5 for EV^Hep^ and n = 5 for *Klb*^HepKO^, *HFD*; n = 6 for EV^Hep^ and n = 5 for *Klb*^HepKO^), (F) plasma liver damage markers AST/ALT (*Chow*; n = 5 for EV^Hep^ and n = 5 for *Klb*^HepKO^, *HFD*; n = 6 for EV^Hep^ and n = 5 for *Klb*^HepKO^), (G) hepatic triglyceride levels (*Chow*; n = 5 for EV^Hep^ and n = 5 for *Klb*^HepKO^, *HFD*; n = 6 for EV^Hep^ and n = 5 for *Klb*^HepKO^) and (H) hepatic phospholipid levels (*Chow*; n = 5 for EV^Hep^ and n = 5 for *Klb*^HepKO^, *HFD*; n = 6 for EV^Hep^ and n = 5 for *Klb*^HepKO^) in *Klb*^HepKO^ mice compared to EV^Hep^ mice following 4 weeks of chow diet or 12 weeks of HFD. Data are presented as mean ± 95% CI in Tukey box- and whisker plots with individual datapoints and histological images. Statistical significance was determined using nonparametric Mann–Whitney testing for the comparison of two groups and indicated as ∗*P* < 0.05, ∗∗*P* < 0.01, ∗∗∗*P* < 0.001, ∗∗∗∗*P* < 0.0001. EV: empty vector, KO: knockout, KLB: beta-klotho, HFD: high-fat diet, H&E: haematoxylin & eosin, AST: aspartate aminotransferase, ALT: alanine aminotransferase.

### 2.4 Untargeted proteomics reveals metabolic consequences of hepatic KLB deletion

To identify potential molecular mechanisms underlying the observed changes in bile acid and hepatic lipid metabolism following hepatic loss of KLB, we performed untargeted proteomics on the livers and plasma of the chow-fed mice, revealing 36 differentially regulated proteins in liver (|log2FC| threshold = 0.378; adj.p-value threshold = 0.05 with Benjamini-Hochberg correction) (Figure 5A–C), whereas no changes were observed in plasma. In line with increased *Cyp7a1* mRNA levels (Figure 2A) upon knockout of *Klb*, we found a 1.7-fold increase in Cyp7a1 protein levels. Apart from proteins involved in bile acid metabolism, also various proteins involved in lipid metabolism were changed, including acetyl-coenzyme A acyltransferase 1B (Acaa1b, 1.7-fold increased), fatty acid elongase 5 (Elovl5, 1.7-fold decreased), and perilipin 2 (Plin2, 1.6-fold decreased). These findings show that in addition to bile acid synthesis, various pathways controlling lipid metabolism, such as fatty acid oxidation, lipoprotein metabolism, and the formation of lipid droplets were dysregulated in KLB-deficient livers. This was further demonstrated in gene set enrichment analysis (GSEA) that also demonstrated the biosynthesis of unsaturated fatty acids as a top-enriched pathway (Figure 5D, E) and the presence of fatty acid elongation as another activated pathway upon knockout of hepatic KLB.

**Figure 5:**
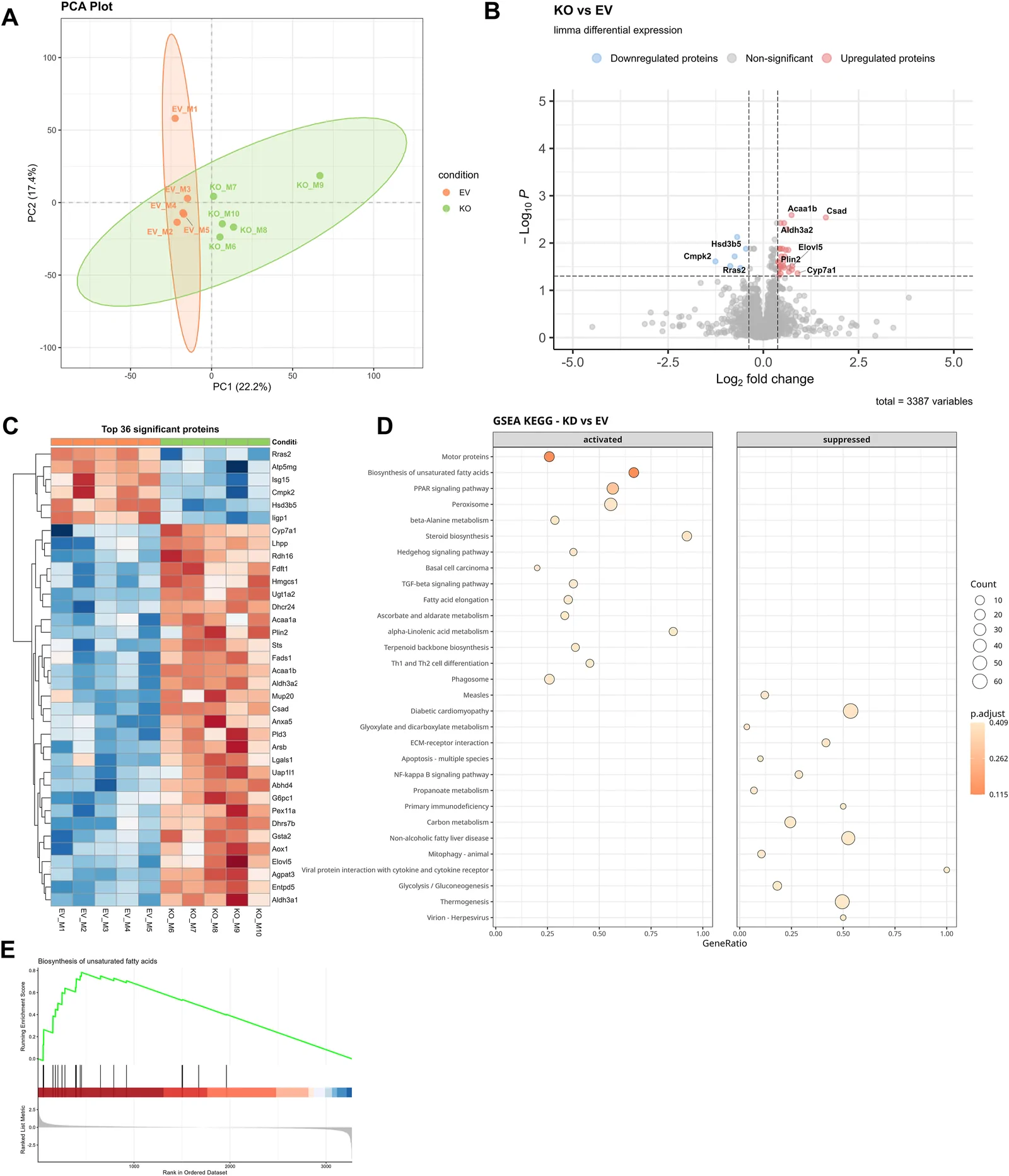
**Loss of hepatic KLB affects bile acid metabolism and hepatic lipid metabolism in chow-fed mice.** (A) Principal component analysis (PCA) revealing clear separation along the first principal component, (B) Volcano plot demonstrating all differentially expressed proteins, (C) Top 30 significantly different proteins, (D) Top 30 significantly different pathways, and (E) Biosynthesis of unsaturated fatty acids as the top affected pathway in *Klb*^HepKO^ mice compared to EV^Hep^ mice following 4 weeks of chow diet. Data are presented as PCA plots, volcano plots, heatmaps, and dot plots. Limma was used to assess differences in protein levels, followed by KEGG pathway analysis. EV: empty vector, KO: knockout, KLB: beta-klotho, PCA: principal component analysis, GSEA: gene set enrichment analysis.

### 2.5 Loss of hepatic KLB alters VLDL metabolism

To investigate how loss of hepatic KLB could lead to increased hepatic lipid content under chow conditions, we measured changes in hepatic lipid fluxes. Hepatic KLB deficiency did not significantly alter total plasma acylcarnitine levels or NADH as a measure of oxidation, nor did it alter *de novo* lipogenesis gene expression or ACC1 levels in chow-fed mice, suggesting that hepatic loss of KLB does not affect fatty acid oxidation or synthesis (Figure 6A, G, Supplementary Figure 3H-I). In contrast, analysis of plasma VLDL, LDL, and HDL fractions using FPLC demonstrated that, only under chow-fed conditions, the relative plasma triglyceride and cholesterol levels in the VLDL fraction were 2.8-fold and 1.9-fold decreased, respectively, in chow-fed *Klb*^HepKO^ mice compared to EV^Hep^ mice (Figure 6B, C). The hepatic TG/ApoB ratio, a proxy for VLDL lipidation, does not seem significantly altered under chow conditions (Figure 6H, Supplementary Figure 3J). Similarly, plasma ApoB levels, which give insight into the total VLDL particle number, show no significantly different levels in chow-fed *Klb*^HepKO^ mice compared to EV^Hep^ mice (Supplementary Figure 3K), indicating no differences in particle number. However, we did observe an increased hepatic TG/PL ratio under chow conditions (Figure 6I), showing that there are less phospholipids available than triglycerides, creating an imbalance in lipoprotein metabolism. Thereby, an altered hepatic TG/PL ratio could contribute to the altered VLDL metabolism we observe in KLB-deficient mice. Upon HFD feeding, changes in plasma VLDL levels and the effects of KLB on hepatic triglyceride accumulation were lost. Likewise, hepatic TG secretion as determined by poloxamer-407 assay under HFD conditions only showed no changes (Figure 6D–F). Thus, these findings suggest that there exists an altered VLDL metabolism alongside increased hepatic triglyceride accumulation following loss of hepatic *Klb* under chow conditions, and these effects do not exist under HFD conditions. Whether the altered VLDL homeostasis contributes to the increased hepatic triglyceride accumulation remains insufficiently characterised.

**Figure 6:**
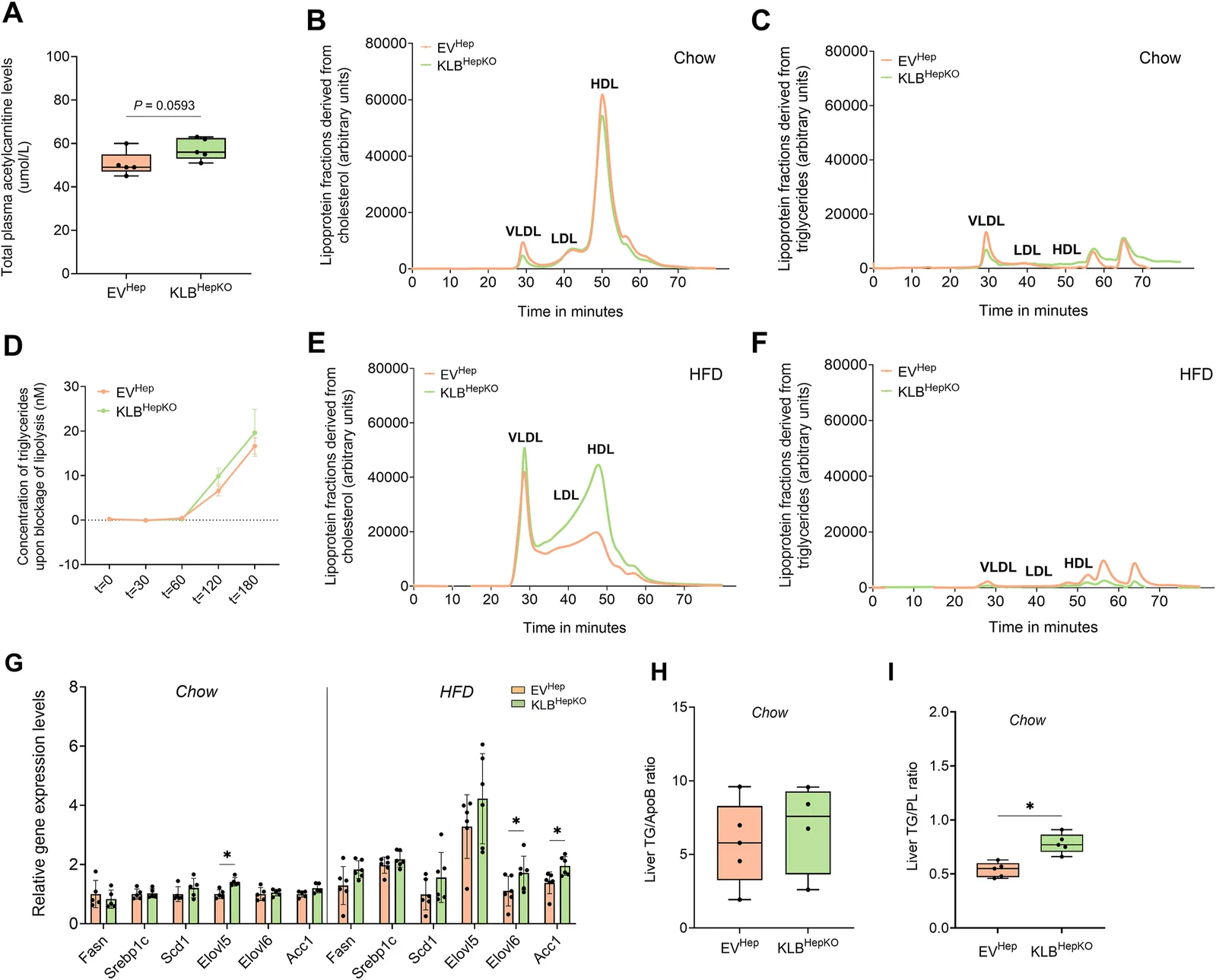
**Hepatic KLB deficiency reduces plasma VLDL levels in chow-fed mice.** (A) Total plasma acylcarnitine levels in *Klb*^HepKO^ mice compared to EV^Hep^ mice under chow-fed conditions. (B) Lipoprotein fraction derived from cholesterol, and (C) Lipoprotein fraction derived from triglycerides and cholesterol in *Klb*^HepKO^ mice compared to EV^Hep^ mice under chow-fed conditions. (D) Concentration of triglycerides upon blockage of lipolysis using poloxamer-407 in *Klb*^HepKO^ mice compared to EV^Hep^ mice under HFD conditions. (E) Lipoprotein fraction derived from cholesterol, (F) Lipoprotein fraction derived from triglycerides, and (G) *de novo* lipogenesis gene expression in *Klb*^HepKO^ mice compared to EV^Hep^ mice under HFD conditions. (H) Liver TG/ApoB ratio in chow-fed *Klb*^HepKO^ mice compared to EV^Hep^ mice. (I) Liver TG/PL ratio in *Klb*^HepKO^ mice compared to EV^Hep^ mice under chow and HFD conditions. Data are presented as mean ± 95% CI in Tukey box- and whisker plots and line diagrams. Chow has n = 4 or 5/group for both EV^Hep^ and KLB^HepKO^, and HFD has n = 6/group for EV^Hep^ and n = 5/group for KLB^HepKO^. Significance is indicated as ∗*P* < 0.05, ∗∗*P* < 0.01, ∗∗∗*P* < 0.001 by Mann–Whitney testing. EV: empty vector, KO: knockout, KLB: beta-klotho.

## 3 Discussion

In this study, we generated a somatic, liver-specific KLB knockout mouse model to delineate the short-term consequences of hepatic KLB loss in adulthood while avoiding the developmental abnormalities associated with germline Klb knockouts [10]. To explore the physiological consequences of hepatic KLB deficiency, we characterized male mice on a chow diet (4 weeks) or HFD (12 weeks). No differences were found in body weight, fat and lean mass, or liver weight between groups, indicating that the observed metabolic changes were not caused by morphometric differences. Consistent with previously described germline *Klb* knockout models, hepatic KLB deficiency increased bile acid synthesis and shifted bile acid composition towards a more hydrophobic bile acid pool under both dietary conditions, thereby strengthening the evidence for a vital role of hepatic KLB in regulating bile acid homeostasis. Notably, loss of hepatic KLB also affected lipid homeostasis, particularly lipoprotein metabolism, characterized by reduced circulating VLDL levels together with marked hepatic triglyceride accumulation under chow-fed conditions. In response to an HFD, these effects were also present up to 8 weeks but disappeared after prolonged HFD feeding.

The increased bile acid synthesis in *Klb*-deficient mice was associated with increased *Cyp7a1* and *Cyp8b1* expression, which is consistent with findings in germline knockout models [10,19,20]. Under physiological conditions, postprandial release of bile acids activates FXR-mediated secretion of FGF15/19 in ileal enterocytes, which acts through the hepatic KLB/FGFR4 complex to transcriptionally repress *Cyp7a1,* the rate-limiting enzyme for production of cholic acid (CA) via the classical pathway. Upon loss of hepatic KLB, *Cyp7a1* is derepressed, causing increased bile acid synthesis [9,25,26]. Under both dietary conditions, hepatic loss of KLB also increased *Cyp8b1* mRNA expression, another enzyme in the classical pathway of bile acid synthesis [24,27]. While CYP7A1 and CYP8B1 protein levels were not quantitatively assessed, we detected greater proportions of CA and DCA upon hepatic loss of KLB. These findings are in line with upregulated *Cyp7a1* and *Cyp8b1* expression, showing that transcriptional changes are supported by functional metabolic consequences. Bile acid synthesis is related to bile acid secretion, which drives bile flow via osmotic diffusion of water into bile [28,29]. Indeed, bile acid synthesis and biliary secretion were more strongly increased under HFD conditions compared to chow conditions, which aligns with previous studies showing that HFD feeding alters *Cyp7a1* and *Cypb81* expression, as well as bile acid composition and bile flow, to support increased lipid intake and absorption [11,30]. Moreover, *Klb*-deficient mice also excreted more cholesterol and phospholipids into bile under HFD conditions, which is in line with the notion that bile acid secretion promotes biliary cholesterol and phospholipid secretion to prevent hepatocellular damage [29] and that KLB-deficient mice are resistant to gallstone formation [8]. The increased bile acid synthesis resulted in increased expression of ileal *Fgf15*, reflecting increased bile acids in the ileum and the activation of ileal FXR. These findings are in line with the reported increase in bile acid synthesis and secretion. Under normal conditions, the murine bile acid pool mainly comprises muricholic bile acids, which are very hydrophilic and, therefore, confer little toxicity [27]. Despite a shift from hydrophilic muricholic acids towards hydrophobic bile acids (CA and DCA), which could potentially exert pro-inflammatory effects on hepatocytes, no changes in liver histology or plasma AST/ALT were observed. Whereas this shift did not induce short-term hepatotoxicity, long-term consequences of a more toxic bile acid pool remain to be determined.

Previous studies in human and rat hepatocytes demonstrated that bile acids can regulate VLDL assembly and secretion, underscoring their role in controlling serum lipid levels [31,32]. Consistent with this, we observed decreased plasma triglyceride levels on chow diet and up to 8 weeks of HFD and increased hepatic triglyceride levels on chow diet in KLB deficient mice, while NEFA levels remained unchanged. While previously reduced plasma triglyceride levels were reported in germline KLB knockout mice [10], lower hepatic triglyceride levels have been reported after 8 weeks of HFD, which was attributed to increased CA to DCA conversion and TGR5-mediated thermogenesis [11]. Although we also observed elevated DCA levels, we observed a trend towards increased hepatic triglyceride accumulation at 12 weeks of HFD rather than a reduction, indicating different responses to HFD feeding in germline versus somatic KLB knockout mouse models. As there is little evidence for liver injury although we do observe accumulation of hepatic triglycerides, this study mainly informs on the early hepatic lipid-handling defects that occur upon loss of hepatic KLB. The transient reduction in plasma triglycerides at 8 weeks of HFD that resolves by 12 weeks suggests that the liver adapts to the fat overload through KLB-independent compensatory mechanisms. It should be noted that plasma triglyceride levels are lower in HFD-fed mice compared to chow-fed mice, which could be attributed to altered lipid handling upon high fat content of the HFD, contrary to the carbohydrate-rich chow diet.

To identify hepatic processes underlying these altered lipid levels, we performed untargeted proteomics and identified increased HMG-CoA reductase (HMGR) levels, which has previously been observed in germline Klb^−/−^ mice [8]. Additional changes in proteins involved in the breakdown (ACAA1B), synthesis (ELOVL5), and storage (PLIN2) of lipids further implicate a role for KLB in hepatic lipid metabolism, especially towards lipid accumulation upon loss of hepatic KLB [[33], [34], [35], [36]].

To further investigate the mechanisms underlying hepatic lipid accumulation, we examined pathways involved in hepatic TG disposal, including fatty acid oxidation and VLDL-TG secretion. Despite the increased hepatic TG levels in KLB-deficient mice, circulating acylcarnitine levels were unchanged, suggesting that hepatic fatty acid oxidation was not substantially altered [37,38]. We therefore considered whether impaired hepatic VLDL-TG metabolism could contribute to the reduced circulating VLDL-TG levels observed in KLB-deficient mice. Under chow-fed conditions, VLDL particle number and lipidation were comparable between KLB-deficient and EV-treated mice, indicating that differences in VLDL particle abundance or lipidation are unlikely to explain the reduction in circulating VLDL-TG. However, KLB-deficient mice displayed an increased hepatic TG/PL ratio, which may reflect changes in hepatic lipid composition or VLDL particle assembly. Such alterations could potentially influence the incorporation of TG into VLDL particles and, consequently, their secretion or subsequent metabolism.

The possibility that hepatic KLB deficiency alters VLDL secretion or hepatic lipid export is consistent with previous findings showing that changes in hepatic Klb expression affect FGF21 signalling and modulate VLDL-TAG secretion [39]. However, our data do not directly establish whether the reduced circulating VLDL-TG levels result from impaired hepatic VLDL secretion, altered lipid export, or changes in VLDL metabolism. Poloxamer-407 experiments performed under HFD conditions did not reveal an apparent defect in VLDL-TG secretion under these dietary conditions, although this does not exclude alterations in VLDL secretion under chow-fed conditions, particularly in light of the altered hepatic lipid composition observed in KLB-deficient mice. Thus, while our findings do not support a major defect in VLDL-TG secretion under HFD conditions, altered VLDL handling under chow-fed conditions still remains a potential contributor to hepatic TG accumulation in KLB-deficient mice.

As effects on hepatic triglyceride levels disappear after 12 weeks of HFD, an altered VLDL metabolism due to loss of hepatic KLB may have been counterbalanced by HFD-stimulated VLDL secretion, thereby neutralizing effects on triglyceride metabolism. Besides, HFD-induced changes in adipose tissue lipid handling could also promote PPARα-dependent fatty acid oxidation but this seems unlikely due to unaltered plasma NEFA levels. Finally, suppression of SREBP1c-driven lipogenesis through increased bile acid signalling could also underlie the transient phenotype under HFD conditions, although hepatic FXR was not activated despite elevated bile acids. However, the underlying compensatory pathways responsible for the transient phenotype under HFD conditions remains to be determined in future studies as well as the temporal dynamics of this compensation occurs, as longitudinal monitoring of plasma lipids is lacking in this study. Moreover, there might be possible secondary effects of altered bile acid homeostasis influencing lipid metabolism, e.g. through altered intestinal lipid absorption or regulation of bile acid-activated receptors. While we could not fully elucidate the contribution of these factors, elevated ileal FGF15 expression in *Klb*-deficient mice suggests ileal FXR activation. While we only detected altered VLDL metabolism in chow-fed KLB-deficient mice and not HFD-fed KLB-deficient mice, despite both having increased Cyp7a1 expression, we anticipate that changes in VLDL metabolism are not mediated by bile acid synthesis pathways. Nonetheless, the extent to which bile acid metabolism affects lipid metabolism or vice versa remains to be further elucidated, e.g. using KLB^−/−^Cyp7a1^−/−^ double knockout mice.

The alterations in bile acid and lipid metabolism observed in KLB-deficient mice are consistent with disrupted FGF19/21 signalling, which we also observed through a loss of Egr1 protein in KLB-deficient mice. This underscores the importance of the liver in mediating the metabolic actions of FGF19/21. This aligns with clinical observations from FGF-based drugs in MASLD/MASH patients that demonstrate reduction of hepatic steatosis and fibrosis, and marked reduction in C4 levels [40,41], suggesting that pharmacological modulation of the hepatic FGF19/21-FGFR-KLB signalling axis contributes to the metabolic improvements reported for FGF-based drugs in clinical studies. The increased bile acid synthesis observed in KLB-deficient mice also supports the therapeutic potential of FGF-based drugs in cholestatic liver disease, where suppression of bile acid production is desirable. As KLB was deleted before HFD feeding in our experimental design, our current data primarily addresses the role of hepatic KLB in the development and progression of diet-induced metabolic dysfunction, rather than establishing a role for KLB in established MASLD/MASH. Future isotope-tracing studies of hepatic KLB function could deepen mechanistic understanding of how FGF19/21 analogs regulate lipid flux and may help explain their therapeutic benefits. Finally, the strong metabolic phenotype associated with hepatic loss of KLB suggests that there may be associations between *KLB* variants and alterations in bile acid and lipid metabolism, which could influence responsiveness to FGF-based therapies.

While this study reports the effects of somatic KLB knockout exclusively in male mice, future studies should also investigate whether these findings are also observed in females. Male mice were used to facilitate comparison with previous studies of germline KLB deficiency, which predominantly use male mice [10,11,13,16], allowing us to distinguish effects associated with developmental loss of KLB from those resulting from somatic KLB deficiency in adulthood. However, findings from this study cannot be generalized to female mice as sex-specific differences in lipogenesis, including potential modulation by oestrogen [42], as well as differences in bile acid homeostasis between male and female mice [8], may influence the metabolic consequence of KLB deficiency. Moreover, the CRISPR/Cas9-based generation of these mouse models carries a potential risk of off-target effects. To address this concern, we generated a second independent somatic KLB mouse model using alternative guide RNAs targeting exon 1 and 2. The similar phenotypes observed in both models support the robustness of our findings and substantially reduce the likelihood that the observed effects are attributable to a model-specific off-target effect, as the two guide RNAs are unlikely to share the same off-target profile. Nevertheless, the possibility of incomplete or mosaic editing should also be acknowledged, as not all hepatocytes may undergo KLB deletion, which would lead to an underestimation of the effects of KLB deficiency.

Taken together, our study provides novel insight into KLB function, independent of its effects on development. In contrast to germline knockout mouse models, somatic loss of hepatic KLB did not impact body weight or liver weight, indicating that these effects are either derived from KLB expression in the brain or adipose tissue, or due to developmental defects, but could also arise after prolonged chow or HFD feeding. As the role of KLB in bile acid metabolism is evident in both germline and somatic mouse models and across dietary conditions, our findings reinforce and replicate earlier evidence identifying KLB as a key regulator of enterohepatic bile acid homeostasis. In addition to its role in bile acid metabolism, we show here a novel role for hepatic KLB in lipid homeostasis, possibly via regulation of VLDL metabolism. These findings contribute to a better overall understanding of KLB function and has implications for the development and interpretation of FGF19/KLB-directed therapies for MASLD and related metabolic diseases.

## 4 Methods

### 4.1 Animal experiments

To generate mice with hepatocyte-specific Cas9 expression, Rosa26-LSL-Cas9 knock-in mice (#026175, The Jackson Laboratory) were crossed with Albumin-Cre mice (#003574, The Jackson Laboratory) expressing Cre recombinase under the control of Albumin (Alb) gene promotor [43]. To blunt hepatic KLB expression, 8–10 weeks old male mice were injected in a randomized manner retro-orbitally with either 0.5 × 10^11^ adeno-associated virus (AAV) particles containing three single guide RNAs (sgRNAs) targeting exon 1 of the mouse *Klb* gene to induce a liver-specific knockout of Klb (KLB^HepKO^) or a matched viral dose containing empty AAV vector (EV^Hep^). Only male mice were used in this study to ensure better comparison to previously reported findings from germline models that used only male mice. To validate the reproducibility of our findings and avoid possible off-target concerns, an independent second mouse model was generated targeting exon 1 and 2 of KLB with different guide RNAs rather than only exon 1. Mice were individually housed in a light- and temperature-controlled facility with a 12 h light/dark cycle at 21 °C with ad libitum access to water and diet. All animal experiments were performed with the approval of the National Ethics Committee for Animal Experiments of the Netherlands, in accordance with relevant guidelines and regulations.

Mice were fed a chow diet (RM1; SDS Diets, Woerden, The Netherlands) for 4 weeks (n = 10) or fed a high-fat diet (HFD) (60% kcal fat rodent diet D12492i; Brogaarden, Lynge, Denmark) for 12 weeks (n = 11). Body weight and appearance of the mice was monitored weekly. In mice fed an HFD, body composition was measured prior to HFD and at 4,8, and 12 weeks after starting a HFD using nuclear magnetic resonance (NMR) MiniSpec (MiniSpec LF90 BCA-analyzer, Bruker). After 8 weeks of HFD, blood was collected from the tail vein to measure plasma lipid levels during diet intervention. After 4 weeks of standard chow diet feeding or 12 weeks of HFD, mice were anesthetized by intraperitoneal injection of hypnorm (1 mL/kg fentanyl-fluanisone) and diazepam (10 mg/kg), followed by gallbladder cannulation as described previously [44]. Bile was collected for 20 min, after which mice were terminated by cardiac puncture under isoflurane anaesthesia, followed by cervical dislocation. The liver was collected, snap-frozen in liquid nitrogen, and stored at −80 °C until further analysis. Blood was drawn by cardiac puncture, and plasma was collected after centrifugation at 1000×*g* for 10 min at 4 °C and stored at −80 °C until further analysis.

### 4.2 Adeno-associated virus generation

Expression cassettes encoding sgRNA sequences targeting exon 1 of *Klb* (sgRNA1: TTACCGGCTTCTCCGGAGAC, sgRNA2: TAAAAAACAGTACGTGAGTC, sgRNA3: TGCTCCGGTCCCAACGCCCC) and targeting both 1 and 2 (sgRNA1: TTGGAGACCGTGTCAAATAT; sgRNA2: CATGCCAAGCAACAAGGTAA; sgRNA3: TAGTCGCCGTCCCCGTGGAT) were synthesized and cloned into an scAAV2 backbone vector using Kpnl and Notl (New England Biolabs), after which sequence verification was performed. HEK293T cells with adenovirus early region 1 encoded within their genome were transfected with the vector, a pAd-deltaF6 helper plasmid (112867, Addgene; a gift from James M. Wilson) and a pAAV2/8 Rep/Cap-encoding plasmid (112864, Addgene; a gift from James M. Wilson) using polyethylenimine (23966-2, Polysciences Inc). An iodixanol gradient was used to isolate the virus particles produced by the cells, followed by spin filter centrifugation with a 100 kDa cut-off (UFC910024, Merck) to concentrate the particles. The AAV genome copies were quantified using qPCR, and virus aliquots were stored at −80 °C until use.

### 4.3 Quantitative real-time PCR

Frozen liver tissue was crushed using a mortar and pestle in liquid nitrogen to obtain liver powder. Liver powder was then homogenized in QIAzol Lysis Reagent (79306, Qiagen) and isolated using the RNeasy Mini kit (74104, Qiagen). Next, 1000 ng of isolated RNA was reverse transcribed into complementary DNA using a mixture of 200 U/μL Moloney Murine Leukemia Virus reverse transcriptase (28025013, Invitrogen), 50 μM random nonamers (R7647, Sigma Aldrich), 10 mM deoxynucleotide triphosphates (11969064001, Roche), 40 U/μL RNAse Out (10777019, Invitrogen), and 0.1 M dithiothreitol (DTT) (28025013, Invitrogen) in buffer solution. cDNA was amplified using Hi-ROX SensiMix™ SYBR green (QT605-05, Bioline). Primers for genes of interest and the housekeeping gene U36B4 were added in a final concentration of 300 nM (Supplementary Table 1). qPCR analysis was performed on the Quantstudio5™ Real-Time PCR System (Applied Biosystems). Gene expression levels were quantified using the standard curve and normalized to the housekeeping gene.

### 4.4 Immunoblotting

Frozen liver powder was homogenized in 0.1% NP40 buffer containing 0.1% Nonidet P-40 (NP40) (21–3277, Sigma Aldrich), 0.4 M NaCl, 10 mM Tris–HCl (pH 8.0), and 1 mM EDTA, supplemented with 1x Complete protease inhibitor (11836145001, Roche) and 1:100 Halt phosphatase inhibitor cocktail (78420, ThermoFisher). Protein concentration was determined using the Pierce™ BCA Assay kit (23225, Thermo Scientific). 30 μg of protein was loaded per lane on 4–15% Mini-Protean TGX Precast Protein gels (4561086, Bio-Rad), which were run at 120 V. Samples were transferred to a 0.2 μm nitrocellulose membrane (170–4159, Bio-Rad) using the Trans-Blot Turbo Transfer System (Bio-Rad) for 10 min at 25 V. Membranes were blocked in 5% BSA in PBS and incubated overnight at 4 °C with 1:1000 diluted primary antibody (Supplementary Table 2). Membranes were washed with tris-buffered saline supplemented with 0.5% Tween-20 (TBS-T) (p2287, Sigma Aldrich), followed by 1 h incubation with 1:2000 diluted secondary antibody (Supplementary Table 2). After washing with TBS-T, proteins were visualized using Supersignal West Pico Plus ECL mix (34578, Thermo Scientific) on the Chemidoc Touch Imaging system and analysed using Image Lab software version 6.0 (Bio-Rad).

### 4.5 Untargeted proteomics

Discovery-based proteomics analyses were done as described previously [45]. In short, in-gel digestion was performed on 50 μg liver homogenates with 500 ng trypsin or 15 μg plasma with 150 ng trypsin. Liquid chromatography mass spectrometry (LC-MS)-based proteomics analyses were performed with an equivalent of 1 μg total protein liver homogenate digest. LC-MS raw data were processed with Spectronaut (version 18.5.231110) (Biognosys) with a Mouse SwissProt database (17141 entries) using the standard settings of the directDIA workflow except the quantification was performed on MS1. For the quantification, local normalization was applied and the Q-value filtering was set to the classic setting without imputation. Pathway enrichment analysis was performed using iPathwayGuide (Advaita Bio).

### 4.6 Histological analysis

Liver tissues were fixed in 4% paraformaldehyde (PFA), embedded in paraffin, and sliced at 4 μm. The liver tissue sections were stained with haematoxylin and eosin (H&E) according to standard protocols. Part of the tissues were also fixed with O.C.T and snap-frozen in liquid nitrogen to generate cryosections. These sections were stained with Oil Red O according to standard protocols. All slides were scanned using a Hamamatsu slide scanner and quantified using ImageJ [46].

### 4.7 Bile acid analysis

Bile acids in faeces, plasma, and bile were analysed by ultra high-performance liquid chromatography tandem mass spectrometry (UHPLC-MS/MS) and gas–liquid chromatography (GC), as described previously [47].

### 4.8 Plasma measurements

Total cholesterol (TC) levels in plasma were determined using a colorimetric assay (113009910026, Diasys) with a cholesterol standard (113009910030, Diasys). Plasma triglyceride (TG) and glycerol levels were measured using a colorimetric assay (157109910026, Diasys) with a Precimat glycerol standard (10166588130, Roche). Non-esterified fatty acids (NEFAs) were determined using a colorimetric assay (157819910935, Diasys) with oleic acid as a standard (157809910065, Diasys). All measurements were performed according to the manufacturer's protocol.

### 4.9 Fast-performance liquid chromatography (FPLC)

TC and TG content of the major lipoprotein classes (VLDL, LDL, and HDL) were measured using FPLC analysis. Briefly, the system consisted of a PU-4180 RHPLC pump and an UV-4075 UV-Vis detector (Jasco). Plasma samples were diluted in PBS and loaded onto a Superose Increase 10/300 GL column (GE Healthcare) for separation of lipoproteins at a flow rate of 0.31 mL/min. A second flow at 37 °C was used to add cholesterol (1489232, Roche) or triglyceride (1187771, Roche) enzymatic reagents at a flow rate of 0.10 mL/min. Peaks were classified as VLDL, LDL, or HDL, after which area-under-the-curve (AUC) data were collected.

### 4.10 Hepatic lipid extraction and determination of hepatic phospholipids, triglycerides, and total and free cholesterol

Lipid extraction was performed on liver homogenates (15% w/v in PBS for chow-fed mice and 2.5% w/v in PBS for HFD-fed mice) using the Bligh and Dyer method [48]. Briefly, liver homogenates were combined with H_2_O and chloroform/methanol (1:2 v/v). After 30 min of incubation, H_2_O and chloroform were added, the mixture was vortexed and centrifuged at 500×*g* for 10 min at room temperature (RT). The organic layer was transferred to a new glass tube and evaporated under a N_2_ stream. Dried lipids were dissolved in 1 mL of chloroform.

For the quantification of hepatic phospholipids, dissolved lipids in chloroform were evaporated, after which 70% HClO_4_ was added. Samples were boiled for 60 min at 160 °C, after which phosphate reagent and 10% w/v ascorbic acid were added. After additional boiling for 10 min, samples were cooled to RT and absorbance was measured at 750 nm to determine phospholipid levels as previously described [49].

For the quantification of hepatic cholesterol, free cholesterol, and triglycerides, the dissolved lipids in chloroform were evaporated and resolubilized in 2% Triton-X-100 in chloroform. The solvent was evaporated under a N_2_ stream, and the dissolved lipids were solubilized in H_2_O. Total hepatic cholesterol (113009910026, Diasys) and triglycerides (57109910917, Diasys) were determined according to the manufacturer's protocol. Free glycerol levels were determined using TEA buffer supplemented with 200 mM phenol, 3 mM 4-aminotipyrine, 80 U/mL glucokinase, 10 U/mL G3PO, and 20 U/mL peroxidase, which was added to the samples, followed by 60 min of incubation at 37 °C and measurement of absorbance at 540 nm. Free cholesterol levels were determined using 75 mM Pipes buffer, 20 mM sodium cholate, 3 mM 4-aminophenazon, 200 mM phenol, 10% Triton X-100, 8000 U/mL peroxidase, 15 U/mL cholesterol oxidase, and demi water, which was added to the samples, followed by 60 min of incubation at 37 °C and measurement of absorbance at 540 nm.

### 4.11 Biliary lipid content

Biliary cholesterol concentrations were measured by GC as described above for faecal neutral sterol measurement following lipid extraction according to Bligh and Dyer [48]. Biliary phospholipids were determined as described [50].

### 4.12 Acylcarnitine profiling

Acylcarnitine profiling was performed using plasma with the addition of acetonitrile and internal standard (l-carnitine-[D3], acetyl-l-carnitine-[D3], propionyl-l-carnitine-[D3], octanoyl-l-carnitine-[D3], and palmitoyl-l-carnitine-[D3]). Samples were centrifuged at 12,000 g for 10 min, collected in glass vials, and analysed by LC-MS/MS as previously described [51].

### 4.13 VLDL-TG production assay

Following a 4-hour fast, mice were intraperitoneally injected with Poloxamer 407 (16758, Sigma–Aldrich) solution in PBS (1 g/kg body weight) to block lipoprotein lipase activity. Blood samples were collected in heparin-coated tubes from the tail vein at time points 0, 30, 60, 120, and 240 min after Poloxamer 407 injection. Plasma was collected after centrifugation at 1000×*g* for 10 min at 4 °C, followed by measurement of triglyceride and glycerol levels.

### 4.14 FAO activity assay

Detection of fatty acid oxidation (FAO) ability in mouse liver was performed using the Fatty Acid Oxidation (FAO) Colorimetric Assay Kit (E-BC-K784-M, Elabscience), according to the manufacturer's protocol. Briefly, roughly 2.5% mouse liver tissue homogenate with known protein concentrations was treated with chromogenic working solution and substrate working solution, followed by 30 min of incubation at 37 °C. Absorbance was determined at 450 nm using a microplate reader. The amount of enzyme in 1 g of protein per 1 min that hydrolyzes the substrate to produce 1 μmol NADH at 37 °C was defined as 1 unit and was determined by calculating the concentration based on the standard curve, followed by correction for the reaction time, the protein concentration per sample, and the dilution factor of the homogenate.

### 4.15 Statistical analysis

Statistical analyses were performed using GraphPad Prism 10.2.3. software. Proteomics data was filtered, logtransformed, normalized, and analysed using limma (Supplementary Figure 4A). Proteins were filtered out if ≥ 2 missing values were reported per condition and duplicates were removed. Logtransformation and quantile normalization was performed on the data (Supplementary Figure 4B-C), followed by differential expression analysis through the limma package (v.3.67.3), KEGG pathway analysis, and gene set enrichment analysis (GSEA) using the enrichplot package (v.1.32.0) [52] and pathview package (v.1.52.0) [53], and the ClusterProfiler package (v.4.20.0) [54]. The volcanoplot was generated using the EnhancedVolcano package (v.1.30.0). The heatmap was generated using the pheatmap package (v.1.0.13). The complete analysis of the proteomics dataset was performed in RStudio (v2026.04.0) using R version 4.6.0.

Given the small sample size per group (n = 5–12), formal tests of normality were considered to have limited power and were therefore not used to determine normality. Instead, distributions were assessed visually, and non-parametric testing was performed for comparisons between KLB-deficient indicating skewness. Accordingly, the non-parametric Mann–Whitney test was used for the primary analyses comparing EV-treated and KLB-deficient mice. Sensitivity analyses using the Welch's t-test yielded consistent results for key comparisons. All data are presented as median ± interquartile range (IQR) and as mean ± min–max with individual data points in box-whisker plots. All authors had access to the study data and had reviewed and approved the final manuscript. Significance was indicated as ∗*P* < 0.05, ∗∗*P* < 0.01, ∗∗∗*P* < 0.001, and ∗∗∗∗*P* < 0.0001.

## CRediT authorship contribution statement

**Alexandra S. Aaldijk:** Writing – review & editing, Writing – original draft, Visualization, Validation, Project administration, Methodology, Investigation, Formal analysis, Data curation, Conceptualization. **Cristy R.C. Verzijl:** Writing – review & editing, Validation, Resources, Project administration, Methodology, Investigation, Data curation, Conceptualization. **Milaine V. Hovingh:** Writing – review & editing, Software, Resources, Methodology, Investigation, Formal analysis. **Nicolette C.A. Huijkman:** Writing – review & editing, Validation, Resources, Methodology. **Marieke Smit:** Writing – review & editing, Validation, Resources, Methodology. **Niels J. Kloosterhuis:** Writing – review & editing, Resources, Methodology, Investigation. **Niels L. Mulder:** Writing – review & editing, Software, Resources, Methodology, Investigation. **Rick Havinga:** Writing – review & editing, Resources, Methodology, Investigation. **Albert Gerding:** Writing – review & editing, Resources, Methodology, Investigation. **Trijnie Bos:** Writing – review & editing, Resources, Investigation. **Mirjam Koster:** Writing – review & editing, Resources, Methodology, Investigation. **Mariska de Graaf:** Writing – review & editing, Software, Resources, Methodology, Investigation, Formal analysis, Data curation. **Justina C. Wolters:** Writing – review & editing, Software, Resources, Methodology, Investigation, Formal analysis, Data curation. **Bart van de Sluis:** Writing – review & editing, Supervision, Resources, Methodology. **Dicky Struik:** Writing – review & editing, Writing – original draft, Supervision, Project administration, Methodology, Investigation, Conceptualization. **Johan W. Jonker:** Writing – review & editing, Writing – original draft, Supervision, Project administration, Methodology, Funding acquisition, Conceptualization.

## Ethics declaration

This study was conducted in accordance with the ARRIVE (Animal Research: Reporting of In Vivo Experiments) guidelines. This study was approved by the IvD.

## Grant support

This study was supported in part by a research grant from Investigator-Initiated Studies Program of Merck Sharp & Dohme Corp to JWJ and a grant from the Netherlands organization for scientific research (Vici grant 016.176.640) to JWJ. The opinions expressed in this paper are those of the authors and do not necessarily represent those of Merck Sharp & Dohme Corp. This project was co-financed by the Ministry of Economic Affairs and Climate Policy by means of the PPP-allowance made available by Health ∼ Holland, Top Sector Life Sciences & Health to stimulate public-private partnerships.

## Declaration of competing interest

The authors declare no conflicts of interest.

## Figures and Tables

**figs1:**
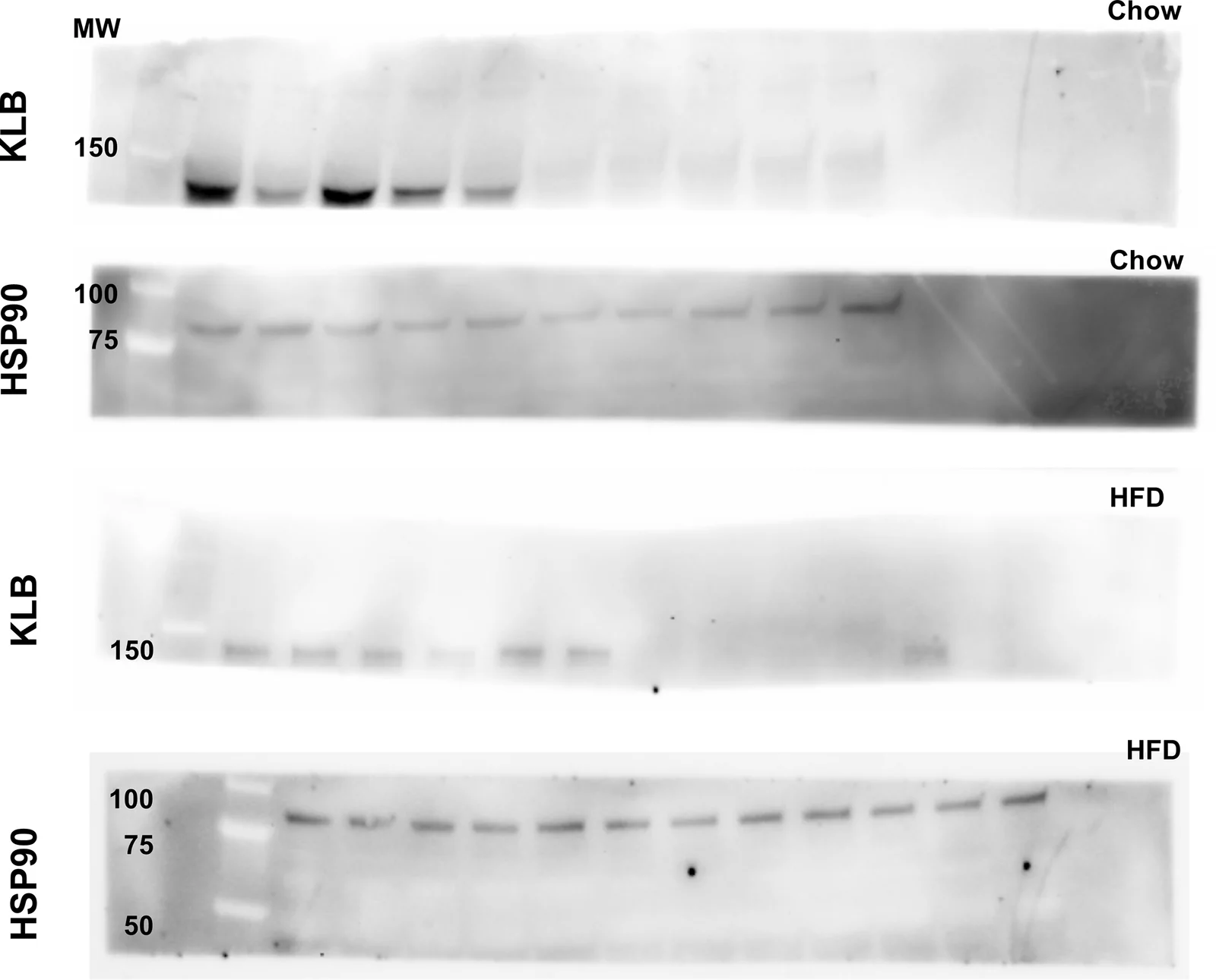
Original uncropped Western Blot images for KLB and HSP90 protein in chow-fed and HFD-fed mice. 30μg of protein was loaded. Molecular weights from the ladder were added on the left side of the blot. KLB protein levels were normalised to HSP90. KLB: beta klotho; HSP90: heatshock protein 90; MW: molecular weight; HFD: high-fat diet.

**figs2:**
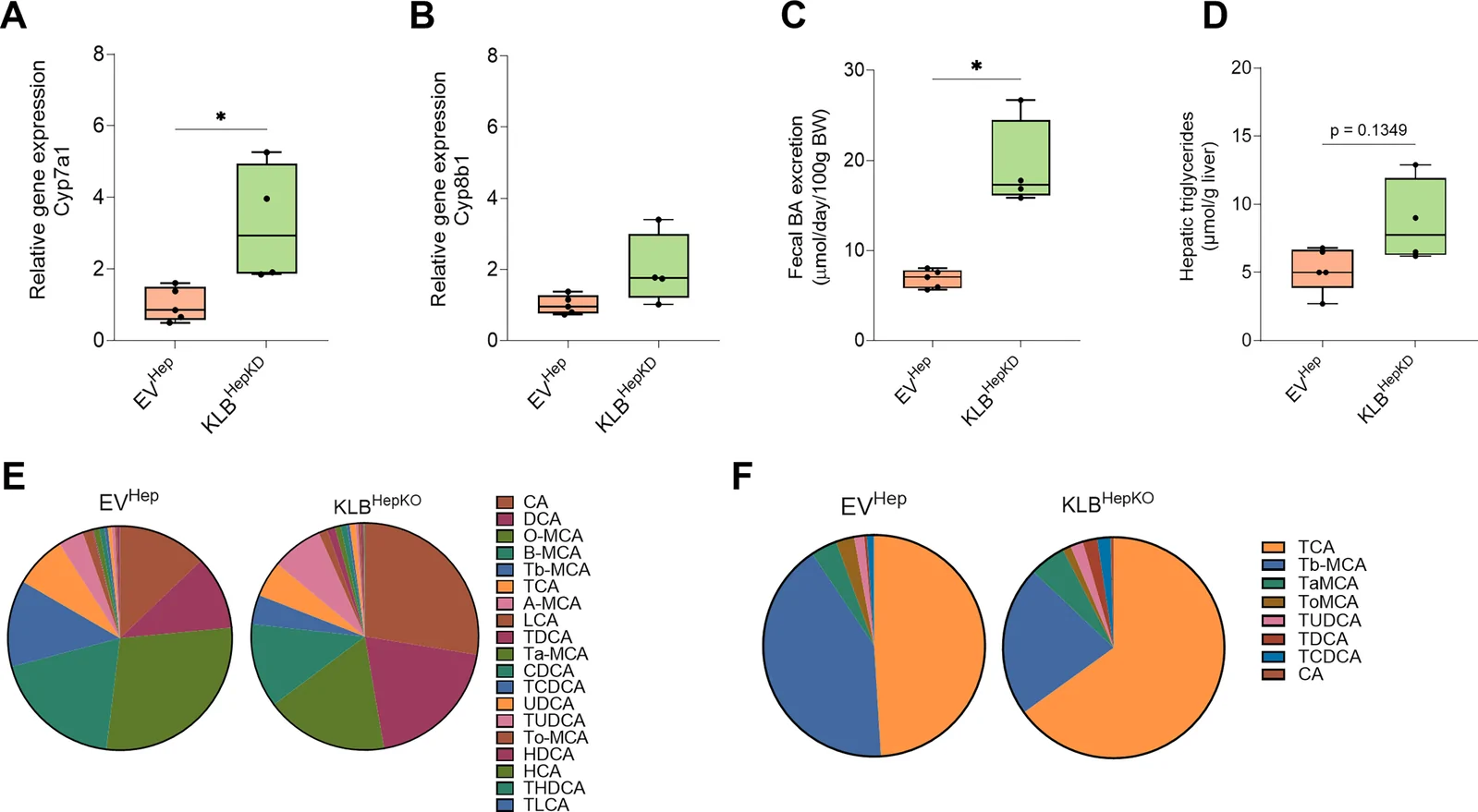
Hepatic KLB deficiency affects bile acid and lipid metabolism in an independent mouse model. (A) Cyp7a1 gene expression, (B) Cyp8b1 gene expression, (C) Fecal bile acid excretion, (D) Hepatic triglyceride levels, (E) Fecal bile acid composition, (F) Biliary bile acid composition in chow-fed KLB^HepKO^ mice compared to EV^Hep^ mice. Data are presented as mean ± min-max in Tukey box- and whisker plots and circle diagrams. Significance is indicated as *P<0.05, P<0.01, ∗∗P<0.001* by Mann-Whitney testing. EV: empty vector; KO: knockout; KLB: beta-klotho.

**figs3:**
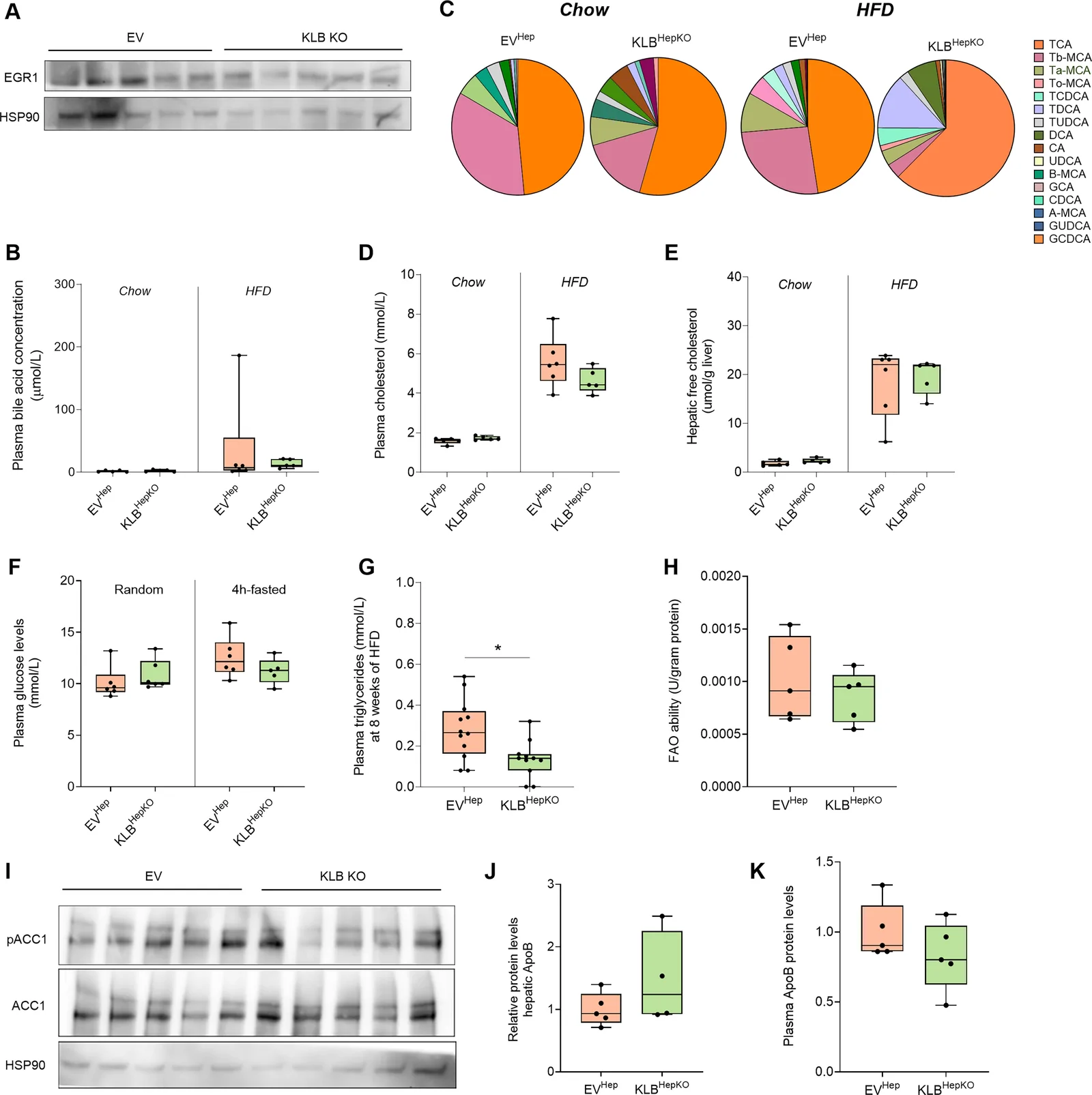
Metabolic parameters in KLB^HepKO^ mice compared to EV^Hep^ under chow-fed or HFD conditions. (A) Hepatic EGR1 protein levels (n=5 for EV^Hep^ and n=5 for Klb^HepKO^), normalized against HSP90, in Klb^HepKO^ mice compared to EV^Hep^ mice following 4 weeks of chow diet. (B) Plasma bile acid concentration (Chow; n=5 for EV^Hep^ and n=5 for Klb^HepKO^, HFD; n=6 for EV^Hep^ and n=5 for Klb^HepKO^), (C) Plasma bile acid composition (Chow; n=5 for EV^Hep^ and n=5 for Klb^HepKO^, HFD; n=6 for EV^Hep^ and n=5 for Klb^HepKO^), (D) Plasma cholesterol levels (Chow; n=5 for EV^Hep^ and n=5 for Klb^HepKO^, HFD; n=6 for EV^Hep^ and n=5 for Klb^HepKO^), and (E) Hepatic free cholesterol levels (Chow; n=5 for EV^Hep^ and n=5 for Klb^HepKO^, HFD; n=6 for EV^Hep^ and n=5 for Klb^HepKO^) in Klb^HepKO^ mice compared to EV^Hep^ mice following 4 weeks of chow diet or 12 weeks of HFD. (F) Plasma glucose levels (n=6 for EV^Hep^ and n=5 for Klb^HepKO^) in Klb^HepKO^ mice compared to EV^Hep^ mice following 12 weeks of HFD. (G) Plasma triglyceride levels at intermediate measurement of plasma extracted from the tail vein (n=12 for EV^Hep^ and n=12 for Klb^HepKO^) in KLB^HepKO^ mice compared to EV^Hep^ mice following 8 weeks of HFD. (H) Fatty acid oxidation ability (n=5 for EV^Hep^ and n=5 for Klb^HepKO^), (I) hepatic phospho-ACC1 and total ACC1 levels (n=5 for EV^Hep^ and n=5 for Klb^HepKO^), all normalized against HSP90, (J) Hepatic ApoB protein levels (n=5 for EV^Hep^ and n=4 for Klb^HepKO^), and (K) plasma ApoB protein levels (n=5 for EV^Hep^ and n=5 for Klb^HepKO^) in Klb^HepKO^ mice compared to EV^Hep^ mice following 4 weeks of chow diet. Data are presented as mean ± 95% CI in Tukey box- and whisker plots with individual datapoints and immunoblots. FAO ability was determined in unit per gram protein, where 1 unit is defined by the amount of enzyme in 1 g protein per 1 minute that hydrolyzes the substrate to produce 1 μmol NADH at 37°C. Statistical significance was determined using nonparametric Mann-Whitney testing for the comparison of two groups and indicated as ∗P<0.05, ∗∗P<0.01, ∗∗∗P<0.001, ∗∗∗∗P<0.0001. EV: empty vector; KO: knockout; KLB: beta-klotho; HFD: high-fat diet; ACC1: Acetyl-CoA-carboxylase 1; FAO: fatty acid oxidation; EGR1: early growth response 1.

**figs4:**
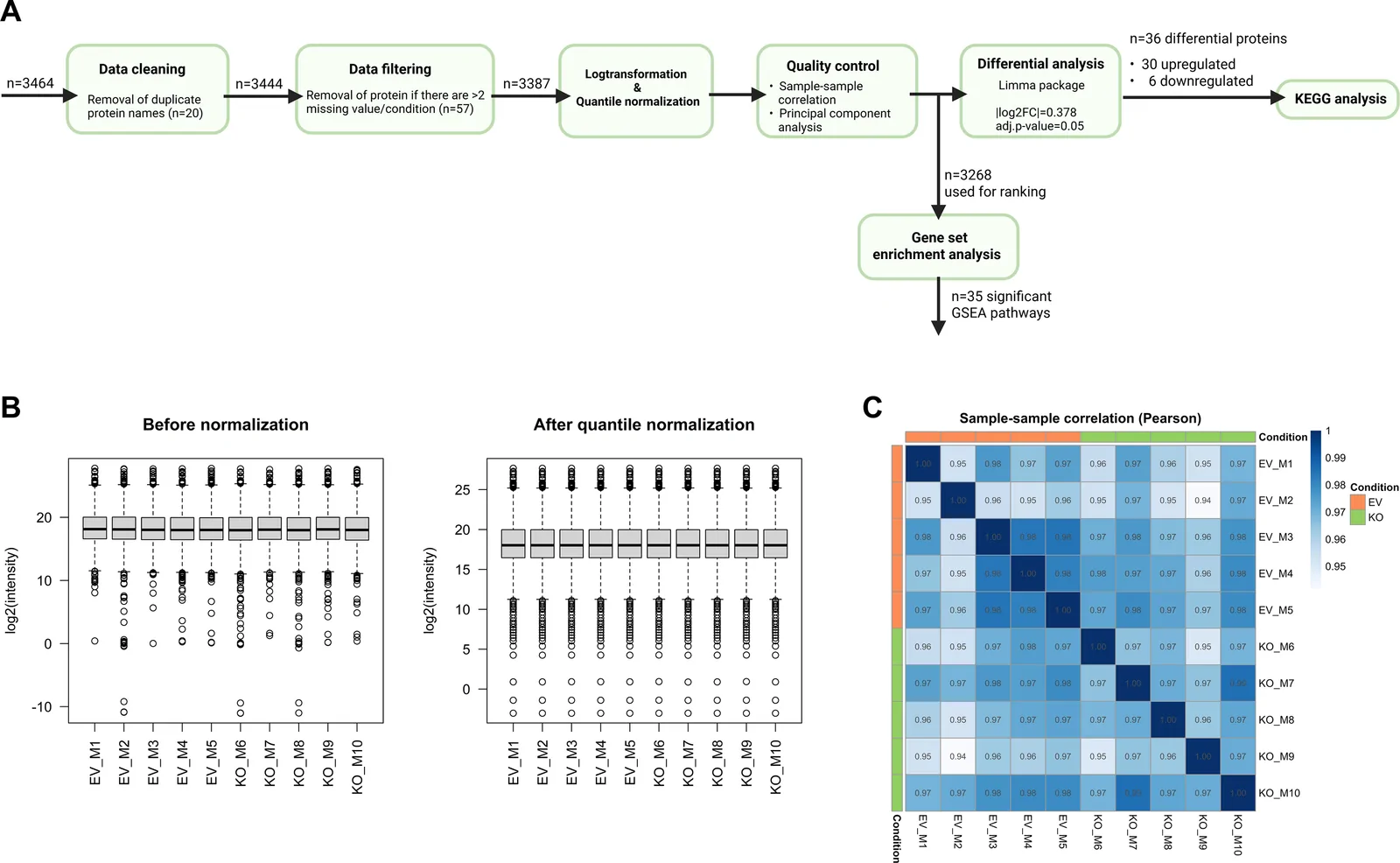
Proteomics workflow of mouse liver tissue from chow-fed KLB^HepKO^ mice compared to EV^Hep^ mice. (A) Workflow including all steps taken, packages and versions used, and cut-offs applied, (B) Quality control on the data before and after quantile normalization, (C) Quality control on the data to verify sample-sample correlation, (D) Barplot with significant pathways derived from KEGG pathway analysis on the 36 reported significantly different proteins, (E) Dotplot with pathways derived from the GSEA analysis. Data are presented as boxplots, barplots, or dotplots. EV: empty vector; KO: knockout; GSEA: gene set enrichment analysis; FC: fold change; KEGG: Kyoto Encyclopedia of Genes and Genomes.

## Data Availability

Data will be made available on request.
