# Supplementary material for: Adult-onset deficiency of β-klotho in the liver reveals a novel role in lipid homeostasis
Source: Mol Metab. 2026 Sep 22;113:102443. doi: 10.1016/j.molmet.2026.102443 (PMC13658126; doi:10.1016/j.molmet.2026.102443)
Supplement: Supplementary file 1 [file mmc1.docx]

**SUPPLEMENTARY MATERIAL**

**Supplementary Table 1. Primer sequences for qPCR.**

| **Gene** | **Forward primer (5’ → 3’)** | **Reverse primer (5’ → 3’)** |
| --- | --- | --- |
| *Cyp7a1* | AGCAACTAAACAACCTGCCAGTACTA | GTCCGGATATTCAAGGATGCA |
| *Cyp8b1* | ACAAGTCGGGCAAGAAGATCC | TTCATCTCGCTGAGGGCAAAG |
| *Cyp27a1* | CATCGCACAAGGAGAGCAATG | AGCCGGGTGATAAAGTCACTG |
| *Cyp7b1* | CTGCGTGACGAAATTGACAG | AAATTCCCAGGCAGACCAAG |

**Supplementary Table 2. Primary and secondary antibodies used for immunoblotting.**

| **Primary antibodies** | | | | |
| --- | --- | --- | --- | --- |
| **Antibody** | **Cat.nr.** | **Company** | **Host** | **Dilution** |
| Anti-KLB | AF5889 | R&D Systems | Goat | 1:1000 |
| Anti-phospho-p44/42 MAPK | 9106S | Cell Signalling | Mouse | 1:1000 |
| Anti-p44/42 MAPK | 9102 | Cell Signalling | Rabbit | 1:1000 |
| Anti-HSP90 | 4874S | Cell Signalling | Mouse | 1:1000 |
| **Secondary antibodies** | | | | |
| Anti-goat IgG HRP | P0449 | Dako | Donkey | 1:2000 |
| Anti-rabbit IgG HRP | P0448 | Dako | Goat | 1:2000 |
| Anti-mouse IgG HRP | P0260 | Dako | Rabbit | 1:2000 |

KLB: beta-klotho, MAPK: mitogen-activated protein kinase, HRP: horseradish peroxidase.

**Supplementary Table 3. Faecal bile acid concentrations in µM with minimum and maximum value of KLB^HepKO^ mice compared to EV^Hep^ mice under chow and HFD conditions.**

| ***Bile acid*** | **Concentration in μM**  **(min – max)** | |
| --- | --- | --- |
|  | ***Chow diet*** | |
|  | **EV^Hep^ (n=5)** | **KLB^HepKO^ (n=5)** |
| CA | 0.201  (0.092 – 0.590) | 1.737  (0.154 – 4.765) |
| TCA | 0.099  (0.088 – 0.110) | 0.236  (0.175 – 0.389) |
| GCA | 0.000  (0.000 – 0.000) | 0.004  (0.000 – 0.008) |
| CDCA | 0.009  (0.007 – 0.012) | 0.047  (0.014 – 0.099) |
| TCDCA | 0.009  (0.005 – 0.014) | 0.018  (0.015 – 0.024) |
| GCDCA | 0.000  (0.000 – 0.000) | 0.000  (0.000 – 0.000) |
| DCA | 0.234  (0.002 – 0.397) | 0.641  (0.000 – 1.421) |
| TDCA | 0.005  (0.000 – 0.007) | 0.021  (0.000 – 0.037) |
| GDCA | 0.000  (0.000 – 0.000) | 0.000  (0.000 – 0.000) |
| UDCA | 0.012  (0.009 – 0.016) | 0.040  (0.013 – 0.077) |
| TUDCA | 0.010  (0.009 – 0.012) | 0.014  (0.011 – 0.018) |
| GUDCA | 0.000  (0.000 – 0.000) | 0.000  (0.000 – 0.000) |
| LCA | 0.023  (0.000 – 0.040) | 0.044  (0.000 – 0.084) |
| TLCA | 0.000  (0.000 – 0.000) | 0.001  (0.000 – 0.003) |
| GLCA | 0.000  (0.000 – 0.000) | 0.000  (0.000 – 0.000) |
| α-MCA | 0.077  (0.054 – 0.121) | 0.372  (0.164 – 0.740) |
| β-MCA | 0.249  (0.188 – 0.342) | 0.376  (0.205 – 0.661) |
| ω-MCA | 0.354  (0.114 – 0.486) | 0.903  (0.602 – 1.313) |
| Tα-MCA | 0.019  (0.009 – 0.030) | 0.070  (0.041 – 0.133) |
| Tβ-MCA | 0.168  (0.142 – 0.200) | 0.163  (0.118 – 0.203) |
| Tω-MCA | 0.011  (0.000 – 0.016) | 0.027  (0.011 – 0.036) |
| HDCA | 0.004  (0.000 – 0.010) | 0.012  (0.000 – 0.026) |
| THDCA | 0.000  (0.000 – 0.000) | 0.001  (0.000 – 0.002) |
| GHDCA | 0.000  (0.000 – 0.000) | 0.000  (0.000 – 0.000) |
| HCA | 0.000  (0.000 – 0.000) | 0.000  (0.000 – 0.000) |
|  | ***High-fat diet*** | |
| CA | 9.330  (2.198 – 23.838) | 182.407  (21.465 – 635.186) |
| TCA | 1.731  (1.172 – 3.309) | 24.068  (1.454 – 101.856) |
| GCA | 0.099  (0.063 – 0.154) | 0.746  (0.117 – 1.424) |
| CDCA | 0.000  (0.000 – 0.000) | 7.565  (0.000 – 22.636) |
| TCDCA | 0.348  (0.268 – 0.447) | 2.743  (0.309 – 11.403) |
| GCDCA | 0.000  (0.000 – 0.000) | 0.077  (0.000 – 0.178) |
| DCA | 40.707  (21.269 – 66.553) | 373.234  (0.398 – 550.844) |
| TDCA | 0.272  (0.152 – 0.529) | 6.849  (0.063 – 24.615) |
| GDCA | 0.089  (0.000 – 0.284) | 0.533  (0.000 – 1.162) |
| UDCA | 0.593  (0.309 – 0.880) | 3.246  (0.928 – 5.433) |
| TUDCA | 0.169  (0.069 – 0.300) | 0.685  (0.078 – 1.708) |
| GUDCA | 0.065  (0.000 – 0.085) | 0.087  (0.078 – 1.708) |
| LCA | 2.692  (1.454 – 4.725) | 18.135  (0.088 – 33.797) |
| TLCA | 0.000  (0.000 – 0.000) | 0.290  (0.000 – 1.205) |
| GLCA | 0.000  (0.000 – 0.000) | 0.027  (0.000 – 0.084) |
| α-MCA | 16.550  (8.538 – 25.987) | 65.709  (12.285 – 96.456) |
| β-MCA | 19.422  (5.877 – 32.752) | 20.087  (11.039 – 42.264) |
| ω-MCA | 15.920  (3.688 – 36.590) | 13.703  (2.504 – 30.229) |
| Tα-MCA | 0.632  (0.489 – 0.981) | 4.420  (0.667 – 15.236) |
| Tβ-MCA | 1.947  (1.214 – 3.935) | 2.899  (0.933 – 7.539) |
| Tω-MCA | 0.433  (0.384 – 0.524) | 0.435  (0.366 – 0.579) |
| HDCA | 0.492  (0.070 – 1.258) | 1.384  (0.000 – 4.094) |
| THDCA | 0.000  (0.000 – 0.000) | 0.043  (0.000 – 0.194) |
| GHDCA | 0.000  (0.000 – 0.000) | 0.000  (0.000 – 0.000 |
| HCA | 0.119  (0.000 – 0.560) | 0.239  (0.000 – 0.635) |

CA: cholic acid, TCA: taurocholic acid, GCA: glycocholic acid, CDCA: chenodeoxycholic acid, TCDCA: taurochenodeoxycholic acid, GCDCA: glycochenodeoxycholic acid, DCA: deoxycholic acid, TDCA: taurodeoxycholic acid, GDCA: glycodeoxycholic acid, LCA: lithocholic acid, TLCA: taurolithocholic acid, GLCA: glycolithocholic acid, α-MCA: alpha-muricholic acid, β-MCA: beta-muricholic acid, ω-MCA: omega-muricholic acid, Tα-MCA: tauro-alpha-muricholic acid, Tβ-MCA: tauro-beta-muricholic acid, Tω-MCA: tauro-omega-muricholic acid, HDCA: hyodeoxycholic acid, THDCA: taurohyodeoxycholic acid, GHDCA: glycohyodeoxycholic acid, HCA: hyocholic acid.

**Supplementary Table 4. Percentage of individual faecal bile acids in KLB^HepKO^ mice compared to EV^Hep^ mice under chow and HFD conditions.**

| ***Bile acid*** | **Percentage (%)** | |
| --- | --- | --- |
|  | ***Chow diet*** | |
|  | **EV^Hep^ (n=5)** | **KLB^HepKO^ (n=5)** |
| CA | 14.02 % | 28.25 % |
| TCA | 6.69 % | 5.31 % |
| GCA | 0.00 % | 0.06 % |
| CDCA | 0.60 % | 0.90 % |
| TCDCA | 0.61 % | 0.45 % |
| GCDCA | 0.00 % | 0.00 % |
| DCA | 15.27 % | 18.86 % |
| TDCA | 0.36 % | 0.64 % |
| GDCA | 0.05 % | 0.04 % |
| UDCA | 0.82 % | 0.79 % |
| TUDCA | 0.69 % | 0.33 % |
| GUDCA | 0.00 % | 0.00 % |
| LCA | 1.51 % | 1.35 % |
| TLCA | 0.00 % | 0.04 % |
| GLCA | 0.00 % | 0.00 % |
| α-MCA | 5.15 % | 7.63 % |
| β-MCA | 16.90 % | 8.05 % |
| ω-MCA | 23.76 % | 20.68 % |
| Tα-MCA | 1.27 % | 1.51 % |
| Tβ-MCA | 11.31 % | 4.06 % |
| Tω-MCA | 0.71 % | 0.66 % |
| HDCA | 0.28 % | 0.37 % |
| THDCA | 0.00 % | 0.03 % |
| GHDCA | 0.00 % | 0.00 % |
|  | ***High-fat diet*** | |
| CA | 8.22 % | 25.11 % |
| TCA | 1.80 % | 2.98 % |
| GCA | 0.09 % | 0.10 % |
| CDCA | 0.00 % | 0.93 % |
| TCDCA | 0.35 % | 0.35 % |
| GCDCA | 0.00 % | 0.01 % |
| DCA | 35.98 % | 49.31 % |
| TDCA | 0.27 % | 0.82 % |
| GDCA | 0.08 % | 0.07 % |
| UDCA | 0.55 % | 0.49 % |
| TUDCA | 0.17 % | 0.11 % |
| GUDCA | 0.06 % | 0.02 % |
| LCA | 2.47 % | 2.41 % |
| TLCA | 0.00 % | 0.03 % |
| GLCA | 0.00% | 0.00 % |
| α-MCA | 14.76 % | 9.33 % |
| β-MCA | 17.69 % | 4.02 % |
| ω-MCA | 13.93 % | 2.44 % |
| Tα-MCA | 0.65 % | 0.59 % |
| Tβ-MCA | 1.97 % | 0.56 % |
| Tω-MCA | 0.43 % | 0.09 % |
| HDCA | 0.43 % | 0.18 % |
| THDCA | 0.00 % | 0.00 % |
| GHDCA | 0.00 % | 0.00 % |

CA: cholic acid, TCA: taurocholic acid, GCA: glycocholic acid, CDCA: chenodeoxycholic acid, TCDCA: taurochenodeoxycholic acid, GCDCA: glycochenodeoxycholic acid, DCA: deoxycholic acid, TDCA: taurodeoxycholic acid, GDCA: glycodeoxycholic acid, LCA: lithocholic acid, TLCA: taurolithocholic acid, GLCA: glycolithocholic acid, α-MCA: alpha-muricholic acid, β-MCA: beta-muricholic acid, ω-MCA: omega-muricholic acid, Tα-MCA: tauro-alpha-muricholic acid, Tβ-MCA: tauro-beta-muricholic acid, Tω-MCA: tauro-omega-muricholic acid, HDCA: hyodeoxycholic acid, THDCA: taurohyodeoxycholic acid, GHDCA: glycohyodeoxycholic acid.

**Supplementary Table 5. Plasma bile acid concentrations in µM with minimum and maximum value of KLB^HepKO^ mice compared to EV^Hep^ mice under chow and HFD conditions.**

| ***Bile acid*** | **Concentration (μM)**  **(min - max)** | |
| --- | --- | --- |
|  | ***Chow diet*** | |
|  | **EV^Hep^ (n=5)** | **KLB^HepKO^ (n=5)** |
| CA | 0.011  (0.000 – 0.054) | 0.070  (0.043 – 0.110) |
| TCA | 0.826  (0.269 – 1.640) | 1.454  (0.304 – 2.820 |
| GCA | 0.000  (0.000 – 0.000) | 0.000  (0.000 – 0.000) |
| CDCA | 0.000  (0.000 – 0.000) | 0.000  (0.000 – 0.000) |
| TCDCA | 0.016  (0.000 – 0.051) | 0.041  (0.000 – 0.099) |
| GCDCA | 0.000  (0.000 – 0.000) | 0.000  (0.000 – 0.000) |
| DCA | 0.025  (0.000 – 0.060) | 0.066  (0.000 – 0.140) |
| TDCA | 0.016  (0.000 – 0.051) | 0.050  (0.000 – 0.109) |
| GDCA | 0.000  (0.000 – 0.000) | 0.000  (0.000 – 0.000) |
| UDCA | 0.000  (0.000 – 0.000) | 0.000  (0.000 – 0.000) |
| TUDCA | 0.038  (0.000 – 0.059) | 0.043  (0.000 – 0.083) |
| GUDCA | 0.000  (0.000 – 0.000) | 0.000  (0.000 – 0.000) |
| LCA | 0.000  (0.000 – 0.000) | 0.000  (0.000 – 0.000) |
| TLCA | 0.000  (0.000 – 0.000) | 0.000  (0.000 – 0.000) |
| GLCA | 0.000  (0.000 – 0.000) | 0.000  (0.000 – 0.000) |
| α-MCA | 0.000  (0.000 – 0.000) | 0.000  (0.000 – 0.000) |
| β-MCA | 0.086  (0.000 – 0.350) | 0.063  (0.037 – 0.078) |
| ω-MCA | 0.000  (0.000 – 0.000) | 0.042  (0.000 – 0.081) |
| Tα-MCA | 0.096  (0.031 – 0.240) | 0.176  (0.042 – 0.357) |
| Tβ-MCA | 0.604  (0.165 – 1.180) | 0.440  (0.094 – 0.876) |
| Tω-MCA | 0.013  (0.000 – 0.066) | 0.041  (0.000 – 0.086) |
| HDCA | 0.000  (0.000 – 0.000) | 0.000  (0.000 – 0.000) |
| THDCA | 0.000  (0.000 – 0.000) | 0.000  (0.000 – 0.000) |
| GHDCA | 0.000  (0.000 – 0.000) | 0.000  (0.000 – 0.000) |
|  | ***High-fat diet*** | |
| CA | 0.087  (0.041 – 0.136) | 0.129  (0.042 – 0.260) |
| TCA | 15.218  (0.633 – 72.205) | 16.476  (3.497 – 54.807) |
| GCA | 0.016  (0.000 – 0.056) | 0.054  (0.000 – 0.110) |
| CDCA | 0.006  (0.000 – 0.037) | 0.033  (0.000 – 0.088) |
| TCDCA | 1.541  (0.046 – 8.411) | 1.624  (0.179 – 6.639) |
| GCDCA | 0.000  (0.000 – 0.000) | 0.013  (0.000 – 0.076) |
| DCA | 0.096  (0.071 – 0.132) | 0.718  (0.093 – 1.412) |
| TDCA | 0.549  (0.052 – 2.696) | 2.416  (0.842 – 5.782) |
| GDCA | 0.000  (0.000 – 0.000) | 0.000  (0.000 – 0.000) |
| UDCA | 0.035  (0.000 – 0.082) | 0.050  (0.000 – 0.088) |
| TUDCA | 1.066  (0.000 – 5.663) | 0.831  (0.098 – 3.370) |
| GUDCA | 0.000  (0.000 – 0.000) | 0.015  (0.000 – 0.087) |
| LCA | 0.000  (0.000 – 0.000) | 0.000  (0.000 – 0.000) |
| TLCA | 0.008  (0.000 – 0.049) | 0.015  (0.000 – 0.092) |
| GLCA | 0.000  (0.000 – 0.000) | 0.000  (0.000 – 0.000) |
| α-MCA | 0.008  (0.000 – 0.049) | 0.019  (0.000 – 0.073) |
| β-MCA | 0.057  (0.000 – 0.255) | 0.021  (0.000 – 0.071) |
| ω-MCA | 0.000  (0.000 – 0.000) | 0.000  (0.000 – 0.000) |
| Tα-MCA | 3.134  (0.220 – 16.274) | 2.449  (0.333 – 11.945) |
| Tβ-MCA | 13.640  (0.336 – 74.599) | 4.137  (0.303 – 22.632) |
| Tω-MCA | 0.613  (0.162 – 2.624) | 0.355  (0.137 – 1.401) |
| HDCA | 0.000  (0.000 – 0.000) | 0.000  (0.000 – 0.000) |
| THDCA | 0.051  (0.000 – 0.307) | 0.024  (0.000 – 0.144) |
| GHDCA | 0.000  (0.000 – 0.000) | 0.000  (0.000 – 0.000) |

CA: cholic acid, TCA: taurocholic acid, GCA: glycocholic acid, CDCA: chenodeoxycholic acid, TCDCA: taurochenodeoxycholic acid, GCDCA: glycochenodeoxycholic acid, DCA: deoxycholic acid, TDCA: taurodeoxycholic acid, GDCA: glycodeoxycholic acid, LCA: lithocholic acid, TLCA: taurolithocholic acid, GLCA: glycolithocholic acid, α-MCA: alpha-muricholic acid, β-MCA: beta-muricholic acid, ω-MCA: omega-muricholic acid, Tα-MCA: tauro-alpha-muricholic acid, Tβ-MCA: tauro-beta-muricholic acid, Tω-MCA: tauro-omega-muricholic acid, HDCA: hyodeoxycholic acid, THDCA: taurohyodeoxycholic acid, GHDCA: glycohyodeoxycholic acid.

**Supplementary Table 6. Percentage of plasma bile acids of KLB^HepKO^ mice compared to EV^Hep^ mice under chow and HFD conditions.**

| ***Bile acid*** | **Percentage (%)** | |
| --- | --- | --- |
|  | ***Chow diet*** | |
|  | **EV^Hep^ (n=5)** | **KLB^HepKO^ (n=5)** |
| CA | 0.35 % | 4.85 % |
| TCA | 48.46 % | 54.44 % |
| GCA | 0.00 % | 0.00 % |
| CDCA | 0.00 % | 0.00 % |
| TCDCA | 0.53 % | 1.13 % |
| GCDCA | 0.00 % | 0.00 % |
| DCA | 2.43 % | 3.98 % |
| TDCA | 0.86 % | 1.98 % |
| GDCA | 0.00 % | 0.00 % |
| UDCA | 0.00 % | 0.00 % |
| TUDCA | 3.30 % | 2.00 % |
| GUDCA | 0.00 % | 0.00 % |
| LCA | 0.00 % | 0.00 % |
| TLCA | 0.00 % | 0.00 % |
| GLCA | 0.00 % | 0.00 % |
| α-MCA | 0.00 % | 0.00 % |
| β-MCA | 3.28 % | 4.28 % |
| ω-MCA | 0.00 % | 3.39 % |
| Tα-MCA | 5.53 % | 6.75 % |
| Tβ-MCA | 34.84 % | 16.07 % |
| Tω-MCA | 0.42 % | 1.12 % |
| HDCA | 0.00 % | 0.00 % |
| THDCA | 0.00 % | 0.00 % |
| GHDCA | 0.00 % | 0.00 % |
|  | ***High-fat diet*** | |
| CA | 1.46 % | 1.02 % |
| TCA | 47.50 % | 61.94% |
| GCA | 0.06 % | 0.29 % |
| CDCA | 0.06 % | 0.24 % |
| TCDCA | 3.30 % | 4.21 % |
| GCDCA | 0.00 % | 0.07 % |
| DCA | 1.99 % | 6.95 % |
| TDCA | 2.39 % | 13.08 % |
| GDCA | 0.00 % | 0.00 % |
| UDCA | 0.39 % | 0.48 % |
| TUDCA | 2.07 % | 2.23 % |
| GUDCA | 0.00 % | 0.08 % |
| LCA | 0.00 % | 0.00 % |
| TLCA | 0.00 % | 0.00 % |
| GLCA | 0.00 % | 0.00 % |
| α-MCA | 0.00 % | 0.14 % |
| β-MCA | 0.16 % | 0.14 % |
| ω-MCA | 0.00 % | 0.00 % |
| Tα-MCA | 9.57 % | 4.25 % |
| Tβ-MCA | 26.09 % | 3.54 % |
| Tω-MCA | 4.91 % | 1.32 % |
| HDCA | 0.00 % | 0.00 % |
| THDCA | 0.03 % | 0.00 % |
| GHDCA | 0.00 % | 0.00 % |

CA: cholic acid, TCA: taurocholic acid, GCA: glycocholic acid, CDCA: chenodeoxycholic acid, TCDCA: taurochenodeoxycholic acid, GCDCA: glycochenodeoxycholic acid, DCA: deoxycholic acid, TDCA: taurodeoxycholic acid, GDCA: glycodeoxycholic acid, LCA: lithocholic acid, TLCA: taurolithocholic acid, GLCA: glycolithocholic acid, α-MCA: alpha-muricholic acid, β-MCA: beta-muricholic acid, ω-MCA: omega-muricholic acid, Tα-MCA: tauro-alpha-muricholic acid, Tβ-MCA: tauro-beta-muricholic acid, Tω-MCA: tauro-omega-muricholic acid, HDCA: hyodeoxycholic acid, THDCA: taurohyodeoxycholic acid, GHDCA: glycohyodeoxycholic acid.

**Supplementary Table 7. Biliary bile acid concentrations in µM with minimum and maximum values of KLB^HepKO^ mice compared to EV^Hep^ mice under chow and HFD conditions.**

| ***Bile acid*** | **Concentration (µM)**  **(min – max)** | |
| --- | --- | --- |
|  | ***Chow diet*** | |
|  | **EV^Hep^ (n=5)** | **KLB^HepKO^ (n=5)** |
| CA | 0.010  (0.000 – 0.052) | 0.046  (0.000 – 0.084) |
| TCA | 15.052  (12.140 – 18.140) | 23.068  (16.140 – 30.800) |
| GCA | 0.000  (0.000 – 0.000) | 0.000  (0.000 – 0.000) |
| CDCA | 0.000  (0.000 – 0.000) | 0.000  (0.000 – 0.000) |
| TCDCA | 0.404  (0.300 – 0.560) | 0.906  (0.328 – 1.350) |
| GCDCA | 0.000  (0.000 – 0.000) | 0.000  (0.000 – 0.000) |
| DCA | 0.000  (0.000 – 0.000) | 0.000  (0.000 – 0.000) |
| TDCA | 0.282  (0.000 – 0.450) | 0.606  (0.000 – 1.174) |
| GDCA | 0.000  (0.000 – 0.000) | 0.000  (0.000 – 0.000) |
| UDCA | 0.000  (0.000 – 0.000) | 0.000  (0.000 – 0.000) |
| TUDCA | 0.519  (0.408 – 0.824) | 0.684  (0.372 – 1.048) |
| GUDCA | 0.000  (0.000 – 0.000) | 0.000  (0.000 – 0.000) |
| LCA | 0.000  (0.000 – 0.000) | 0.000  (0.000 – 0.000) |
| TLCA | 0.000  (0.000 – 0.000) | 0.000  (0.000 – 0.000) |
| GLCA | 0.000  (0.000 – 0.000) | 0.000  (0.000 – 0.000) |
| α-MCA | 0.000  (0.000 – 0.000) | 0.000  (0.000 – 0.000) |
| β-MCA | 0.017  (0.000 – 0.084) | 0.000  (0.000 – 0.000) |
| ω-MCA | 0.000  (0.000 – 0.000) | 0.000  (0.000 – 0.000) |
| Tα-MCA | 2.102  (1.842 – 2.720) | 3.384  (2.460 – 4.360) |
| Tβ-MCA | 13.020  (9.620 – 20.200) | 8.000  (6.300 – 10.480) |
| Tω-MCA | 0.802  (0.000 – 1.190) | 1.136  (0.670 – 1.614) |
| HDCA | 0.000  (0.000 – 0.000) | 0.000  (0.000 – 0.000) |
| THDCA | 0.011  (0.000 – 0.056) | 0.035  (0.000 – 0.104) |
| GHDCA | 0.000  (0.000 – 0.000) | 0.000  (0.000 – 0.000) |
|  | ***High-fat diet*** | |
| CA | 0.075  (0.037 – 0.181) | 0.054  (0.037 – 0.092) |
| TCA | 9.151  (5.944 – 13.090) | 16.396  (13.965 – 19.533) |
| GCA | 0.007  (0.000 – 0.039) | 0.046  (0.040 – 0.049) |
| CDCA | 0.000  (0.000 – 0.000) | 0.000  (0.000 – 0.000) |
| TCDCA | 0.439  (0.339 – 0.526) | 0.993  (0.702 – 1.313) |
| GCDCA | 0.000  (0.000 – 0.000) | 0.000  (0.000 – 0.000) |
| DCA | 0.040  (0.039 – 0.041) | 0.042  (0.038 – 0.048) |
| TDCA | 0.312  (0.136 – 0.457) | 3.200  (2.209 – 4.597) |
| GDCA | 0.000  (0.000 – 0.000) | 0.000  (0.000 – 0.000) |
| UDCA | 0.000  (0.000 – 0.000) | 0.007  (0.000 – 0.036) |
| TUDCA | 0.472  (0.236 – 0.736) | 0.614  (0.322 – 0.866) |
| GUDCA | 0.000  (0.000 – 0.000) | 0.000  (0.000 – 0.000) |
| LCA | 0.000  (0.000 – 0.000) | 0.000  (0.000 – 0.000) |
| TLCA | 0.000  (0.000 – 0.000) | 0.022  (0.000 – 0.039) |
| GLCA | 0.000  (0.000 – 0.000) | 0.000  (0.000 – 0.000) |
| α-MCA | 0.006  (0.000 – 0.036) | 0.007  (0.000 – 0.036) |
| β-MCA | 0.000  (0.000 – 0.000) | 0.000  (0.000 – 0.000) |
| ω-MCA | 0.000  (0.000 – 0.000) | 0.000  (0.000 – 0.000) |
| Tα-MCA | 1.901  (1.118 – 3.241) | 1.325  (1.141 – 1.611) |
| Tβ-MCA | 6.589  (2.897 – 12.618) | 1.087  (0.792 – 1.509) |
| Tω-MCA | 0.414  (0.214 – 0.659) | 0.163  (0.137 – 0.188) |
| HDCA | 0.000  (0.000 – 0.000) | 0.000  (0.000 – 0.000) |
| THDCA | 0.000  (0.000 – 0.000) | 0.000  (0.000 – 0.000) |
| GHDCA | 0.000  (0.000 – 0.000) | 0.000  (0.000 – 0.000) |

CA: cholic acid, TCA: taurocholic acid, GCA: glycocholic acid, CDCA: chenodeoxycholic acid, TCDCA: taurochenodeoxycholic acid, GCDCA: glycochenodeoxycholic acid, DCA: deoxycholic acid, TDCA: taurodeoxycholic acid, GDCA: glycodeoxycholic acid, LCA: lithocholic acid, TLCA: taurolithocholic acid, GLCA: glycolithocholic acid, α-MCA: alpha-muricholic acid, β-MCA: beta-muricholic acid, ω-MCA: omega-muricholic acid, Tα-MCA: tauro-alpha-muricholic acid, Tβ-MCA: tauro-beta-muricholic acid, Tω-MCA: tauro-omega-muricholic acid, HDCA: hyodeoxycholic acid, THDCA: taurohyodeoxycholic acid, GHDCA: glycohyodeoxycholic acid.

**Supplementary Table 8. Percentage of individual biliary bile acids in KLB^HepKO^ mice compared to EV^Hep^ mice under chow and HFD conditions.**

| ***Bile acid*** | **Percentage (%)** | |
| --- | --- | --- |
|  | ***Chow diet*** | |
|  | **EV^Hep^ (n=5)** | **KLB^HepKO^ (n=5)** |
| CA | 0.02 % | 0.09 % |
| TCA | 46.72 % | 60.92 % |
| GCA | 0.00 % | 0.00 % |
| CDCA | 0.00 % | 0.00 % |
| TCDCA | 1.25 % | 2.39 % |
| GCDCA | 0.00 % | 0.00 % |
| DCA | 0.00 % | 0.00 % |
| TDCA | 0.88 % | 1.60 % |
| GDCA | 0.00 % | 0.00 % |
| UDCA | 0.00 % | 0.00 % |
| TUDCA | 1.61 % | 1.81 % |
| GUDCA | 0.00 % | 0.00 % |
| LCA | 0.00 % | 0.00 % |
| TLCA | 0.00 % | 0.00 % |
| GLCA | 0.00 % | 0.00 % |
| α-MCA | 0.00 % | 0.00 % |
| β-MCA | 0.05 % | 0.00 % |
| ω-MCA | 0.00 % | 0.00 % |
| Tα-MCA | 6.52 % | 8.94 % |
| Tβ-MCA | 40.41 % | 21.13 % |
| Tω-MCA | 2.49 % | 3.00 % |
| HDCA | 0.00 % | 0.00 % |
| THDCA | 0.04 % | 0.09 % |
| GHDCA | 0.00 % | 0.00 % |
|  | ***High-fat diet*** | |
| CA | 0.42 % | 0.23 % |
| TCA | 48.61 % | 68.34 % |
| GCA | 0.03 % | 0.19 % |
| CDCA | 0.00 % | 0.00 % |
| TCDCA | 2.49 % | 4.10 % |
| GCDCA | 0.00 % | 0.00 % |
| DCA | 0.24 % | 0.18 % |
| TDCA | 1.76 % | 13.49 % |
| GDCA | 0.00 % | 0.00 % |
| UDCA | 0.00 % | 0.03 % |
| TUDCA | 2.45 % | 2.52 % |
| GUDCA | 0.00 % | 0.00 % |
| LCA | 0.00 % | 0.00 % |
| TLCA | 0.00 % | 0.09 % |
| GLCA | 0.00 % | 0.00 % |
| α-MCA | 0.03 % | 0.03 % |
| β-MCA | 0.00 % | 0.00 % |
| ω-MCA | 0.00 % | 0.00 % |
| Tα-MCA | 9.66 % | 5.52 % |
| Tβ-MCA | 31.79 % | 4.59 % |
| Tω-MCA | 2.52 % | 0.68 % |
| HDCA | 0.00 % | 0.00 % |
| THDCA | 0.00 % | 0.00 % |
| GHDCA | 0.00 % | 0.00 % |

CA: cholic acid, TCA: taurocholic acid, GCA: glycocholic acid, CDCA: chenodeoxycholic acid, TCDCA: taurochenodeoxycholic acid, GCDCA: glycochenodeoxycholic acid, DCA: deoxycholic acid, TDCA: taurodeoxycholic acid, GDCA: glycodeoxycholic acid, LCA: lithocholic acid, TLCA: taurolithocholic acid, GLCA: glycolithocholic acid, α-MCA: alpha-muricholic acid, β-MCA: beta-muricholic acid, ω-MCA: omega-muricholic acid, Tα-MCA: tauro-alpha-muricholic acid, Tβ-MCA: tauro-beta-muricholic acid, Tω-MCA: tauro-omega-muricholic acid, HDCA: hyodeoxycholic acid, THDCA: taurohyodeoxycholic acid, GHDCA: glycohyodeoxycholic acid.
